# Potentially Toxic Elements in Water, Sediments and Fish from the Karstic River (Raša River, Croatia) Located in the Former Coal-Mining Area

**DOI:** 10.3390/toxics11010042

**Published:** 2022-12-31

**Authors:** Zorana Kljaković-Gašpić, Ankica Sekovanić, Tatjana Orct, Dora Šebešćen, Elena Klasiček, Davor Zanella

**Affiliations:** 1 Analytical Toxicology and Mineral Metabolism Unit, Institute for Medical Research and Occupational Health, Ksaverska Cesta 2, 10000 Zagreb, Croatia; 2 Department of Biology, Faculty of Science, University of Zagreb, Rooseveltov trg 6, 10000 Zagreb, Croatia

**Keywords:** inorganic pollutants, metal(loid)s, ICP-MS, European eel (*Anguilla anguilla*), Italian chub (*Squalius squalus*), Prussian carp (*Carassius gibelio*), Italian barbell (*Barbus plebejus*), Adriatic roach (*Rutilus aula*), flathead grey mullet (*Mugil cephalus*), muscle

## Abstract

The assessment of the environmental quality of a sensitive karst aquatic system under the centuries-long anthropogenic influence of the coal mining industry is important for both improving the quality of water resources and protecting aquatic wildlife and human health. In this study, we investigated the anthropogenic impact on the aquatic environment of the upper and middle course of the Raša River through the analysis of a suite of metal(loid)s in three aquatic compartments (water, sediment, fish) using inductively coupled plasma mass spectrometry (ICP-MS). Concentrations of inorganic constituents in water were low, while the chemical composition of stream sediments mainly reflected the geological background of the area, indicating the origin of metal(loid)s from predominantly natural sources. Although comparison with PEC-Q values indicated that existing sediment quality conditions could pose a threat to benthic organisms with regard to Cr and Ni, the constant vertical profiles of these elements suggested their natural origin from the weathering of flysch. Element levels in the muscle of targeted fish species were in accordance with the values typical for low-contaminated freshwater systems, while levels of Cd, Pb and Hg were mostly below the European regulatory limits for toxic elements in foods, indicating that the low concentrations of most contaminants in muscles of fish from the Raša River do not present a risk to humans or other consumers. The obtained data indicated a generally low contamination status of the western part of the Raša River basin with regard to the analyzed inorganic elements.

## 1. Introduction

Istria, the largest Croatian peninsula, known for the unique beauty of both its shoreline and hinterland, natural attractions [1], diversity of habitats and great plant and animal biodiversity [2,3], attracts over 20 million tourists and visitors every year [4]. Although Istria’s landscape is usually perceived as pristine, with clean and unspoiled nature, until recently the entire region had been subjected to ecological changes [5,6,7,8,9] driven by centuries-long coal mining and utilization [10]. In the Labin basin, which was the most important and biggest coal mining district in Istria, approximately 40 million tons of coal had been excavated by 1999, when coal mining and coal combustion activities ceased. This coal, which is characterized by high amounts of organic sulfur and increased levels of several metal(loid)s [10,11], powered two local coal-fired thermal power plants (TPP Vlaška and TPP Plomin) as well as local households, which resulted in the airborne pollution of local soils with various organic and inorganic pollutants [5,6].

On the western side, the Labin Basin is bordered by the Raša River, one of the most significant surface watercourses of the Istrian peninsula. The river and its drainage area have typical geochemical and sedimentological characteristics of a karstic region, such as intensive weathering of source rocks, intermittent torrents and underground flows and karst springs, which are responsible for the downstream transport and sedimentation of eroded suspended material [12]. In addition to those natural processes, the distribution of inorganic elements in the Raša River watercourse could also be affected by different potential anthropogenic sources, such as agriculture, illegal landfills, industrial facilities, waste water runoff from the settlements and, above all, fossil fuel combustion. Considering the history of coal mining in Istria, the anthropogenic impact on the eastern part of the Raša River drainage area, encompassing five mining towns (Labin, Štrmac, Vinež, Krapan, Raša), the Krapan valley, the Krapan brook, the estuary (i.e., the lowest course of the Raša River) and Raša Bay, has been extensively studied addressing a range of contamination issues resulting from former mining activities. Several of these studies have investigated the occurrence and distribution of potentially toxic elements in natural spring waters, seawater, wastewater and coal-mine discharge [7,8], contaminated soils [8,13], vegetables [8,13], birds [13], wild boar [9], contaminated aquatic [13,14] and marine sediment [9], marine fish [9] and marine mussels [9]. Some also investigated the presence of volatile organic compounds (BTEX) in water samples [8] and polycyclic aromatic hydrocarbons (PAHs) in the soil [6] or evaluated potential radioactivity and cytotoxicity in abandoned coal mine discharge [7]. However, except for the pioneering work of Frančišković-Bilinski et al. [15], the impact of former long-term mining activities and potential impact of two thermal power plants (TPP Vlaška and TPP Plomin) on the western part of the Raša River drainage area, encompassing the upper and central course of the Raša River before confluence with the Krapan brook, has never been systematically investigated. Additionally, a multielement analysis of macro and trace elements in biota from the Raša River has not yet been conducted in the entire basin, although sports and recreational fishing in the river, especially in its estuary, which is recognized as a special natural habitat, has a long tradition and is currently regulated by nature protection legislation [16] with the aim of the sustainable management of biological resources.

Given the above stated facts, the main objectives of this study were: (1) to investigate the distributions of selected major and trace elements in the water, sediments and muscle of six fish species from the upper and middle course of the Raša River with the aim of identifying their potential sources and (2) to provide information on the overall water and sediment quality in the upper and middle course of the Raša River. Since no studies previously conducted in the wider area of the Raša River valley have concomitantly evaluated all of the parameters measured here, the overall goal of this study was to define the contamination status of the aquatic environment in the upper and middle course of the Raša River with regard to metal(loid)s, which in turn should represent a foundation for future biomonitoring.

## 2. Materials and Methods

### 2.1. Site Description and Sampling Strategy

**Figure 1:**
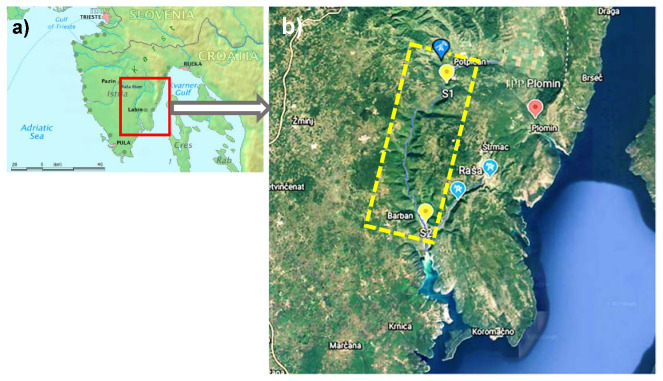
Map of the study area. (**a**) Istrian Peninsula with Raša River (Map modified from Wikipedia), (**b**) Western part of the Raša River basin (yellow rectangle marked with a dashed line), with locations of sampling sites on the Raša River (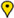), together with locations of former mines in Raša, Labin and Tupljak (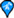) and Thermal Power Plant in Plomin (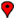) (Map modified from Google Earth).

The Raša River and its estuary are located at the southeastern part of the Istrian peninsula, Croatia (Figure 1). The investigated watercourse of the river Raša represents the western and southwestern border of the wider Labin region, known for its rich deposits of superhigh-organic-sulfur coal. The hydrological regime of the river is typically karstic and characterized by short-term high-discharge events. In the upper part of the drainage area, Eocene flysch sediments predominate, while the central part of the drainage area is dominated by the river flow resulting from the confluence of several streams. The valley floor is covered with quaternary alluvial deposits, while the flanks of the valley are composed of upper Cretaceous limestones [12,17]. A detailed overview of the climatic, pedological, geographical, geological and hydrogeological characteristics of the area were presented previously [7,8,10,11,14].

Samples of water, sediment and fish were collected at two sites in the Raša River (Figure 1); the first (S1) is in the upper course of the river near the Podpićan settlement and the abandoned mining pit Tupljak, which was the last of all shafts to be closed in 1999, with the Ćepić field in the background, and the second (S2) is located 6 km upstream of the Raša River estuary before the confluence with the Krapan brook, which brings water from the direction of former coal mines in Raša and Labin.

Subsurface water samples for the analysis of macro and trace elements were collected in June 2020 using a counter-current hand-grab sampler. Samples without prior filtration for the analysis of total acid leachable metals were immediately acidified to pH < 2 with concentrated ultrapure HNO_3_, stored in a portable refrigerator and transferred to the laboratory where they were stored at 4 °C until analysis.

Samples of river sediments for the analysis of macro and trace elements at both stations were also collected in June 2020. Samples were taken by a scuba diver using pre-cleaned acrylic corers (5 cm inner diameter, length up to 20 cm, depending on site). Immediately after sampling, the cores were frozen and stored at −20 °C until further treatment. In the laboratory, sediment samples were defrosted at room temperature, sliced into 1 cm (0–5 cm depth), 2.5 cm (5–10 cm depth in sediment) and 5 cm (10–20 cm depth) long samples, frozen and freeze-dried (CD 13-2&CD3056 HETOSIC; HETO Ltd., Gydevang, Denmark).

Fish samples were collected within two consecutive sampling campaigns during June of 2020. In total, 104 individuals of 6 fish species, determined according to the key of Kottelat and Freyhof [18], were collected at 2 different locations in the Raša River (Figure 1, Table 1). Not all species were available at all locations. Because of their living and eating habits, samples of European eel (*Anguilla* (Linnaeus, 1758)) and Italian chub (*Squalius squalus* (Bonaparte, 1837)) were collected at both locations. On the other hand, samples of Prussian carp (*Carassius gibelio* (Bloch, 1782), Italian barbell (*Barbus plebejus*, Bonaparte, 1840) and Adriatic roach (*Rutilus aula* (Bonaparte, 1841)) were found exclusively at location S1, while samples of the flathead grey mullet (*Mugil cephalus* (Linnaeus, 1758)) were found exclusively at location S2. Fish were collected by standard ichthyological methods using a 9.5 kW Briggs & Stratton electro-fishing unit, placed in polyethylene bags, transferred to the laboratory, and stored at −18 °C until analysis. Before dissection, fish were thawed at room temperature for 1 hour. The total length and body weight were measured for each individual fish and samples of dorsal muscle tissue were dissected using a ceramic knife. Composite samples were prepared from dorsal muscles of small sized fish to obtain sufficient sample mass, while larger specimens were treated individually. In total, we collected 21 individual samples and 17 composite samples of dorsal muscles, and their basic characteristics are presented in Table 1. Prior to analyses, all samples were freeze-dried (CD 13-2&CD3056 HETOSIC; HETO Ltd., Gydevang, Denmark) and homogenized in a Mixer Mill MM 400 (Retsch, Haan, Germany) using procedures described earlier [19].

### 2.2. Digestion Procedure and Element Analysis

Macro and trace elements in the collected water samples were analyzed directly, without prior dilution using inductively coupled plasma mass spectrometry (ICP-MS) on an Agilent 7500cx (Agilent Technologies, Tokyo, Japan) instrument according to the working conditions presented in Table S1. Collision gases (helium and hydrogen) were used to remove interference, while internal standard solution containing 3 µg L^−1^ of Ge, Rh, Tb, Lu and Ir was used to correct for instrumental drifts and plasma fluctuations. Four standard certified reference materials (NIST SRM 1643e, NIST SRM 1643f, NIST SRM 1641e, NRCC SLRS-5) were analyzed as part of quality control. A list of the analyzed macro and trace elements and LODs for individual elements in water is given in Table S2. The accuracy for most of the analyzed elements in the referent water samples was within ±10% of the certified values, with recoveries ranging from 90% (Ni) to 113% (Ag).

Dry sediment samples (~0.100 g) were wet-digested with a combination of hydrofluoric acid (0.6 mL HF, 48%, Merck; extrapure) and nitric acid (3.0 mL HNO_3_, 65%, p.a., Merck; purified by quartz sub-boiling distillation using the Milestone SubPUR system) in an UltraCLAVE IV digestion system (Milestone Srl, Sorisole, Italy) using an application note for digestion of sediments (Table S3). The digest was diluted with ultrapure water to 30 mL. All samples were prepared in duplicate. All of the analyzed elements were quantified with inductively coupled plasma mass spectrometry (ICP-MS) using an Agilent 7500cx (Agilent Technologies, Tokyo, Japan) according to the working conditions presented in Table S1. Prior to analysis, sediment samples were diluted 20-fold with a solution containing 1% (*v*/*v*) HNO_3_ and 3 µg L^−1^ of internal standards (Ge, Rh, Tb, Lu and Ir) (SCP Science, Quebec, Canada). The LODs ranged from 0.002 mg kg^−1^ dry matter (dm) for U to 45.9 mg kg^−1^ dm for Fe (individual LOD values are given in Table S2). Several standard certified reference materials (NIST SRM 2709, NRCC MESS-3, IAEA SL-1, IAEA-405, PT-SL1) were analyzed as part of quality control. The accuracy for most of the analyzed elements in the referent soil samples was within ±10% of the certified values, with recoveries ranging from 92% (Cr) to 110% (U).

Biological samples (~0.250 g of muscle tissue) were weighed in Teflon vessels and digested with purified concentrated nitric acid and ultrapure water (3:2) in a microwave system (UltraCLAVE IV, Milestone Srl, Sorisole, Italy) using an application note for digestion of biological materials (Table S3). Nitric acid (HNO_3_, 65%, p.a., Merck) was purified using quartz sub-boiling distillation system (SubPUR, Milestone, Sorisole, Italy). After digestion, samples were adjusted to 6 mL with ultrapure water (GenPure, TKA Sytem GmbH, Niederelbert, Germany) and stored at 4 °C until analysis. All of the elements were analyzed by ICP-MS (7500cx, Agilent Technologies, Tokyo, Japan) under similar conditions as described above for macro and trace element analysis in sediment. Biological samples were diluted 7.5-fold with a solution containing 1% (*v*/*v*) HNO_3_ and 3 µg L^−1^ of internal standards (Ge, Rh, Tb, Lu and Ir) (SCP Science, Quebec, Canada). The LODs for fish tissue, expressed per kilogram of wet mass (wm), ranged from 2 ng kg^−1^ wm for Cr to 0.232 mg kg^−1^ wm for Mo (individual LOD values are given in Table S3). Six standard certified reference materials (IAEA-350, IAEA-407, DORM-2, IAEA-436, BCR 185R, BOVINE LIVER 1577a) were analyzed as part of quality control. The accuracy for most of the analyzed elements in referent biological samples was within ±9% of the certified values, with recoveries ranging from 91% (B) to 110% (Pb).

### 2.3. Statistical Analysis

Statistics and visualization were performed using TIBCO Statistica^®^ software, version 14.0.0.15 (TIBCO Software Inc., Palo Alto, CA, USA). Based on the examination of normal score plots of residuals, variables were transformed to achieve normality prior to statistical analysis when necessary. The associations between pairs of contaminants were explored using Pearson’s correlation (r, p). Differences between locations were tested using Mann–Whitney U test (z, P) on original data, while differences between species were tested using Kruskall–Wallis H test. The obtained values were statistically significant at *p* < 0.05. Factor analysis (FA) was used to investigate the factors controlling the spatial distribution of macro and trace elements in sediments of the Raša River system. For the purpose of calculating Se/Hg molar ratios, Se and Hg levels were first divided by the molar weight (78.96 and 200.59 g/mol, respectively).

### 2.4. Pollution Evaluation and Risk Estimation

The pollution level of metal(oid)s in the sediments was assessed using enrichment factor (EF), geo-accumulation index (*Igeo*) and pollution load index (PLI). Since sediment contamination assessments require background concentrations for uncontaminated sediments, in the absence of adequate data for the sediments of the Istrian flysch rivers and considering that the sediments in the Raša River originated mainly from the erosion of the flysch and flysch-like deposits in the drainage area [12,17,20], in this study we used the well-defined geochemical background values of elements in Istrian flysch-derived soils [20,21] as a non-contaminated analogue to calculate the above listed indices for the Raša River sediments, while for elements that were not measured by Peh et al. [20,21] we used either global average values for stream sediment reported by Turekian and Wedepohl [22] or the average values for the Raša River sediment reported by Frančišković-Bilinski [15].

The enrichment factor (EF), which is widely used to quantify the levels of potential contamination of sediments by metal(loid)s, was calculated using the following equation:

EF = (C_i_/C_Al_)_sample/_(C_i_/C_Al_)_background_ (1)

where (C_i_/C_Al_) sample and (C*i*/C_Al_) background is the ratio between metal(loid) *i* and Al in the sediment sample and background sample, respectively. The categories of contamination based on EF values are summarized in Table S4.

The geo-accumulation index, which is extensively used to evaluate pollution level of metal(loid)s in sediment, was calculated using the following equation:

*I_geo_* = Log_2_ (Ci/(1.5 × Bi)) (2)

where Ci and Bi are the concentration of metal(loid) *i* in the sediment sample and its corresponding background value in sediment, respectively, while 1.5 is the factor compensating the background data (correction factor) due to lithogenic effects. The pollution grades based on *Igeo* values are given in Table S4.

Pollution load index (PLI), proposed by Tomlinson et al. [23] as the geometric average of individual pollution indexes of metal(loid)s determined in sediment, was calculated as follows:

(3) $$\mathrm{PLI}=\sqrt[{n}]{C1/B1\;\times \;C2/B2\times \ldots \times Ci/Bi}\;$$

where *Ci* and *Bi* are the concentration of metal(loid) *i* in the sediment sample and its corresponding background value in sediment, while n is the number of elements determined in sediment. The degrees of sediment pollution with respect to the PLI index are also given in Table S4.

The ecological risk of metal(loid)s in the Raša River sediments was accessed using the sediment quality guidelines (SQGs) and ecological risk index (RI) proposed by Hakånson [24]:

(4) $$\mathrm{RI}=\sum_{i=1}^{n}E_{r}^{i}=T_{r}^{i}\;\times \;(C_{s}^{i}/C_{n}^{i})$$

where $C_{s}^{i}$ and $C_{n}^{i}$ is the content of metal(loid) *i* in the sediment and its background value of elements in Istrian flysch-derived soils [15,20,22,25]; $E_{r}^{i}$ is the ecological risk factor of metal(loid) *i;* and $T_{r}^{i}$ is the toxic response coefficient of metal(loid), which is 30 for Cd, 40 for Hg, 10 for As, 5 for Pb, Co, Ni, and Cu, 2 for V and Cr and 1 for Ba, Mn and Zn [24,25]. The ecological risk grades based on the values of $E_{r}^{i}$ and RI are displayed in Table S5.

The potential toxicity of multiple metal(loid)s in the sediments was evaluated using probable effect concentration quotient (PEC-Q) methodology [26], which enables an overall estimation of the possible risk posed by the simultaneous exposure of organisms to several potentially toxic elements. PEC-Q values were calculated using the following equation:

PEC-Q = (ΣC_El/_PEC_El_)_/_n (5)

where C_El_ indicates concentration of element (mg kg^−1^ dw) in sediment, PEC_El_ signifies the corresponding PEC value and *n* stands for the total number of measured elements in sediment sample for which the PEC values are defined (in this case *n* = 8).

The influence of location on metal(loid)s bioaccumulation in fish muscle was estimated by calculating the individual mean multi-elemental bioaccumulation index (IMBI):

(6) $$\mathrm{IMBI}=(\overset{n}{\underset{i=1}{\sum }}C_{i}/C_{i-\max})/n$$

where n is the total number of analyzed metal(loid)s, *C_i_* is the individual concentration of metal(loid) *i*, and *C_i-max_* is the maximum observed concentration of metal(loid) *i* [27].

## 3. Results and Discussion

### 3.1. Element Levels in Water

The average concentrations of the analyzed elements (total acid leachable metals) in the collected water samples are shown in Table 2, along with relevant published data and water quality guidelines. Although obtained element levels were higher than the values reported for the pristine Plitvice Lakes National Park water system [19], the values were still low and in accordance with the average values for Croatian stream waters [28] and with values previously measured in the Raša River estuary [14] or natural freshwater spring (Fonte Gaja) located downstream from the town of Raša [7]. In terms of compliance with various international water quality standards, the concentrations of priority hazardous substances (Cd, Hg) and priority substances (Ni, Pb), as defined by the Water Framework Directive [29], were significantly lower than the proposed maximum allowable concentrations (MAC) for European inland surface waters. Additionally, the concentrations of all potentially toxic elements (PTEs) were significantly lower than the strict Canadian water quality guidelines for the protection of aquatic wildlife [30] (Table 2), indicating that the aquatic system of the upper and middle reaches of the Raša River is not significantly loaded with toxic elements.

### 3.2. Element Levels in Sediments

**Figure 2:**
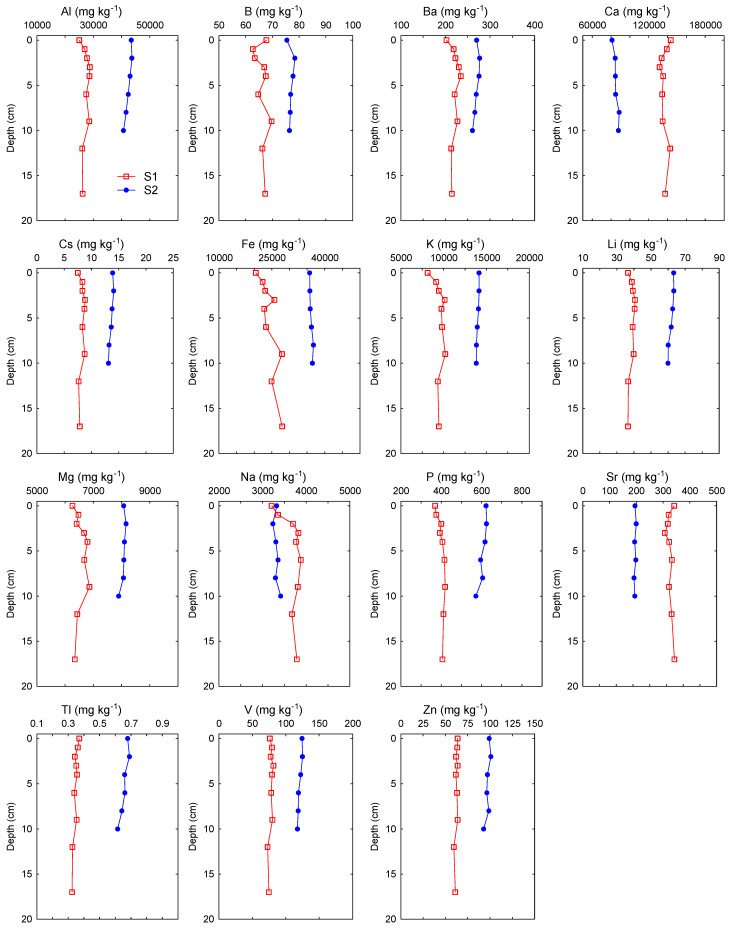

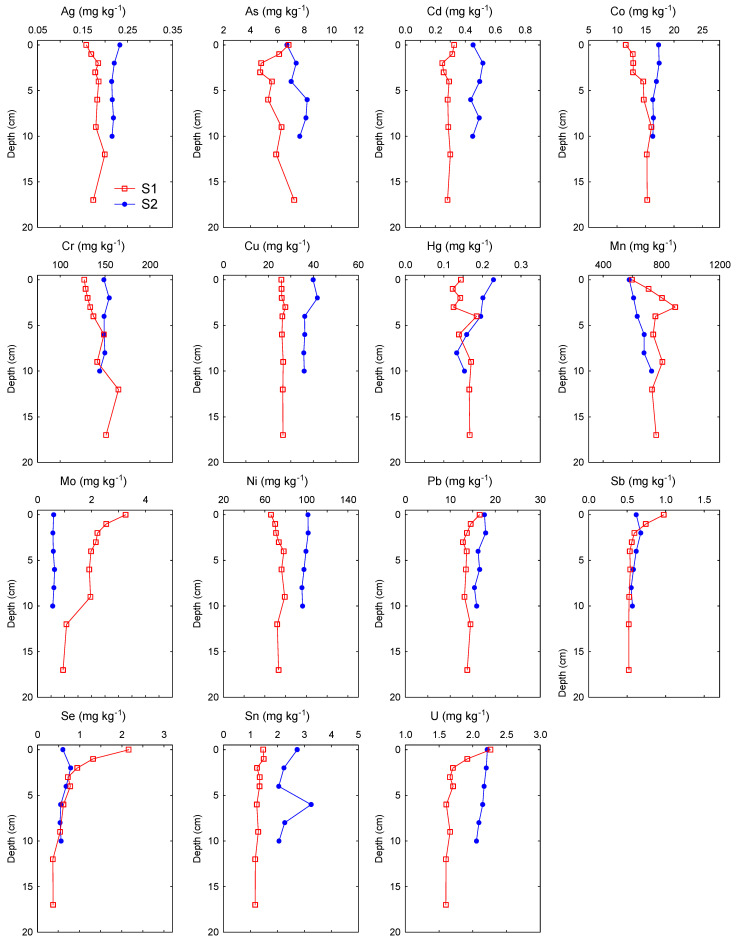
Vertical profiles of major and trace elements in sediment cores from the two locations on the Raša River, collected in June 2020.

The average concentrations of 30 elements in the bulk fraction (<2 mm) of the Raša River sediments are listed in Table 3, while their vertical profiles are presented in Figure 2. In general, the average concentrations of elements in the upper and middle course of the Raša River were slightly higher than the data previously obtained for the freshwater sediments of Raša River or Istrian rivers in general [15], similar or slightly higher than the values for the Raša River estuarine sediments [14], similar or lower than the average values for soils in geographically similar localities such as Raša region soils [21,31] and Plomin control soils [5], and of the same order of the magnitude as the world median data for stream sediment [32]. Furthermore, the obtained data for Cu, Hg, Se and Zn and partially for Cd and Pb were significantly lower than the data for sediment from two channels which used to receive wastewater from separation of washing of coal and/or municipal waste waters from three mining towns and coal mine effluents [8,13], indicating that, in general, sediments from the upper and middle course of the Raša River are not significantly contaminated with anthropogenic elements and are in accordance with the background composition of the surrounding flysch-derived soils.

Considering the history of coal mining in the Labin Basin, an area known for long-term mining of coal rich in various inorganic elements (Cd, Cr, Hg, Mo, Pb, Se, U, V) [8,11], we expected that there might be some locational differences in the Raša River sediments, with levels potentially higher at downstream locations close to the confluence of the Raša River and Krapan stream results of the non-parametric statistical tests showed that most of the analyzed elements, except for Cr, Se, Sb and Hg, significantly differed between locations (Table 3). Most of the elements (Al, As, B, Ba, Cd, Co, Cs, Cu, Fe, K, Li, Mg, Ni, P, Pb, Sn, U, V, Tl, Zn) were elevated in the lower course of the river at location S2. In contrast, certain elements of geogenous (lithogenous) origin (Ca, Na, Mn, Mo, Sr) were significantly higher at location S1, located close to the source of the river and Čepić field, where intensive weathering of the source rocks occurs. For all elements at location S2 and most elements at location S1, variability of the concentrations was rather small (0.9–19.9% at location S2; 2.0–14.6% at location S1), indicating relatively constant element levels during the studied period. Higher variations at station S1 were recorded only for Se (25%), P (35%) and Na (65%). The vertical profiles of the elements in the sediment gave us an even better insight into the state of pollution of the analyzed sediments with inorganic elements (Figure 2). The vertical profiles of conservative lithogenic elements (Al, Fe, Li, K) were rather uniform at both locations, indicating a relatively constant terrigenous input. Similarly, a number of other lithogenic (Ba, Ca, Mg, Na, P, Sr) and trace elements (Ag, B, Cd, Cr, Cs, Cu, Ni, Tl, V, Zn) also showed uniform distribution along the depth profiles at both locations, which indicated a predominately geogenic (natural) origin of these elements over the time scale covered by the sediment cores. Only the concentrations of Mo, Sb, Se and U were elevated in the surface layer (0–5 cm) at location S1, and of Hg in the surface layer (0–5 cm) and Sn in subsurface layer (5–7.5 cm) at location S2, indicating a possible anthropogenic influence.

In order to investigate this issue in more detail, we created a matrix of correlations between elements depending on the location (Table S6). As could be expected, most lithogenic elements (B, Ba, Ca, Cs, Fe, K, Li, Mg, P, Sr, Tl, V) and some trace elements (Ag, Cd, Cu, Sn, Zn) were highly correlated with Al (in all cases r > 0.84). Correlations of Ca, Mo and Sr with Al were negative, reflecting an association of Sr with the carbonate sediment fraction. Since Al is the major constituent of alumosilicates, particularly clay minerals, which are widely accepted as excellent indicators of terrigenous input in the aquatic sediments, high correlations of most elements with Al indicate that these elements are of natural, terrigenous origin, i.e., from the weathering of the source rocks (marl from flysch deposits).

The influence of different factors on the distribution of the analyzed elements in the Raša River sediments was explored using principal component analysis (PCA). PCA extracted three factors with eigenvalues > 1 and accounted for just over 94% of the total variance (Table S7,). Factor 1 was characterized by a strong negative loading of Ca (r = −0.955) and positive loading of Al (r = 0.952) and Li (r = 0.951), suggesting differentiation of sediments under the influence of carbonates and alumosilicates. This factor, which explained 63% of the variation, can be explained as a lithogenic factor indicating terrigenous influence, mostly contributed by the Raša River. The second factor, explaining 16.0% of the data variability, included strong negative effects of Mn and Na and strong positive effects of Sb (r = 0.906) and moderate effects of Se (r = 0.758), U (r = 0.755) and Pb (r = 0.728). This factor included elements whose concentrations either increased (Mn, Na) or decreased (Sb, Se, U, Pb) with depth in the top 5 cm at location S1, while the vertical distribution at location S2 was uniform and did not change with depth. The third factor, explaining 15.1% of the variation, included strong positive effects of Cr, Hg and Co, whose concentrations slightly increased with depth, and a moderate negative effect of Mo, whose concentration significantly decreased with depth in the top 10 cm at location S1. Overall, our results showed that clay fraction (alumosilicates) control the geochemistry of most elements in the Raša River sediments, indicating that most trace elements in sediments are primarily of terrigenous origin and are not altered anthropogenically.

Regarding the sediment quality guidelines (SQG) for metals in freshwater ecosystems [33,34,35] (Table 3), which were designed to protect benthic organisms and to assess the quality of the sediment, concentrations of Fe, Cr, Mn and Ni at both locations and of Cu and Hg in location S2 exceeded the threshold effect concentrations (TEC) (TEC_Fe_ = 2%; TEC_Cr_ = 43.4 mg kg^−1^; TEC_Cu_ = 31.6 mg kg^−1^; TEC_Hg_ = 0.180 mg kg^−1^; TEC_Mn_ = 460 mg kg^−1^; TEC_Ni_ = 22.7 mg kg^−1^), while concentrations of As and Cr at both locations and of Cu and Hg in location S2 exceeded the Interim Freshwater Sediment Quality Guidelines (ISQG) values (ISQG_As_ = 5.9 mg kg^−1^ dw; ISQG_Cr_ = 37.3 mg kg^−1^ dw; ISQG_Cu_ = 35.7 mg kg^−1^ dw; ISQG_Hg_ = 0.170 mg kg^−1^ dw). However, most of the measured values for the above mentioned elements were lower than the probable effect concentrations (PEC) (PEC_As_ = 33 mg kg^−1^ dw; PEC_Cu_ = 149 mg kg^−1^ dw; PEC_Hg_ = 1.06 mg kg^−1^ dw; PEC_Pb_ = 128 mg kg^−1^ dw; PEC_Zn_ = 459 mg kg^−1^ dw) [35], except for Cr and Ni, whose values at both locations exceeded PEC values of 111 mg kg^−1^ dw and 48.6 mg kg^−1^ dw, respectively. That is why the resulting PEC-Q values for both locations (calculated using Equation (6)), which we used to access potential toxicity of those seven potentially toxic elements (PTEs), exceeded the critical value of 0.34 [36], characteristic for areas having a high potential for acute toxicity to amphipods or benthic community impairment. Elevated PEC-Q values, derived mostly from high Ni and Cr content in sediments, indicated that there is a potential ecological risk posed by the simultaneous presence of PTEs in sediments at these locations. However, although the obtained values indicated that sediments in the Raša River could be anthropogenically affected, rather constant Cr and Ni concentrations profiles with depth at both locations (Figure 2), which were also similar to profiles of Co, Mg and Fe (e.g., the elements with which they are commonly associated with Earth’s crust) [31], suggested that the high Cr and Ni contents in sediments are probably of the natural origin (i.e., from weathering of flysch areas with well-developed drainage network [20]). To be more precise, high concentrations of Cr (up to 163 mg kg^−1^ dw) were found in soils from the Raša region [20,21,31], which also partly supports the assumption that Cr also reflects the geological background of the catchment area. Concentrations of other elements for which guideline values exist (Ag, Cd, Co, Pb, Sb and Zn) were lower than the TEC and ISQG values in all of the sediments from the Raša River except for Se, whose value was higher than the TEC value (2 mg kg^−1^ dw) only in the uppermost centimeter at location S1. Our results indicated that the probability that the presence of these metal(loid)s in sediments of the upper and middle course of the Raša River would cause adverse effects on sediment-dwelling organisms was very small.

Further investigation of the sediment quality at the studied sites was conducted through calculation of different sediment quality indices (EF, *I_geo_*, PLI) (Tables S8–S10) as interpretive tools developed to enable us to distinguish between the origin (i.e., natural vs. anthropogenic) of metals in sediments. Hence, most of the values for EF were indicative of deficiency of normal enrichment for almost all of the analyzed variables. Moderate enrichment was observed for Li at both locations, for Ca, Cr, Sr and U at location S1, and for Se in the top 2 cm at location S1 (EF = 2.64–4.66) (Table S8). The apparent enrichment of Ca and Sr in sediment is probably a consequence of the fact that in this study the elements were analyzed in a fraction <2 mm in which carbonate fractions were preserved, in contrast to the data for world average values in a stream sediment analyzed in a fraction <0.63 µm from which strontium-rich carbonate components were removed. The calculated results of *Igeo* indices for metal(loid)s (Table S9) were mostly negative, suggesting an absence of notable trace element pollution in the analyzed sediment samples. However, the maximum values of Li and Cs, primarily in location S2, showed a slight accumulation of these lithogenic elements. Furthermore, although the values of PLI at location S1 (0.832–0.911) were somewhat lower than values at location S2 (1.006–1.073) (Table S10), all of the PLI values were lower than or close to 1, indicating unpolluted sediment. Values at location S2 were higher, probably because of the sedimentation of fine–coarse particles containing higher concentrations of metal(loid)s closer to the estuary [14,37]. Based on the concentrations of elements in sediments, their vertical profiles and calculated sediment indices (EF, *Igeo*, PLI), it can be concluded that the chemical composition of the analyzed stream sediments of the Raša River at both sites mostly reflects the geological and hydrogeological background of the catchment and that sediments are not significantly polluted with inorganic elements.

The ecological risk of metal(loid)s in the Raša River sediments was assessed using calculated *E_r_^i^* and RI values (Table S11). The mean *E_r_^i^* values of the elements from the upper and middle course of the Raša River decreased in the order Cd > Hg > As > Ni~Co~Cu > Pb > Cr > Ba > Zn–Mn. In terms of spatial distribution, the individual *E_r_^i^* values of most elements and overall RI values were higher at the downstream location S2. Of all *E_r_^i^* values, only the value for Cd at location S2 was higher than 40, indicating moderate potential ecological risk of Cd at this location, while other individual *E_r_^i^* values at both locations indicated low potential for ecological risk. The largest contributors to RI were Cd (38.7%) and Hg (27.3%), followed by As (7.8%), while other metal(loid)s contributed with 0.9–5% to the total RI. The obtained RI values (RI_S1_ = 84–101; RI_S2_ = 122–140) were lower than 150, indicating low ecological risk at the investigated locations, as suggested by Håkanson [24].

### 3.3. Element Levels in Fish

In this study, we examined metal(loid) levels in six species in order to investigate the possible influence of trophic level and vertical feeding position of fish on contaminant loads in fish, and we chose fish muscles as the target tissue because they provide information on potential risks to the fish themselves as well as to the consumers. The average values of 22 macro and trace elements, Se:Hg molar ratios and IMBI values in muscle tissue of six fish species from the Raša River are summarized in Table 4. One of our initial hypotheses was that element levels would reflect trophic levels and feeding behavior (listed in Table 1), since trophic-level differences in element levels were reported for a number of contaminants, especially for mercury [38,39,40,41,42,43,44,45,46,47,48,49]. In this study, we found differences between species for all of the analyzed elements, except for Ag, Pb and V, but in most cases, they were not high. Hence, mass fractions of Cd, Cr, Na and Se were highest in eels, and mass fractions of Ca, Mg, Mn, Pb, Sr, U, V and Zn were highest in Adriatic roach, while those of As, Cu, Fe, K, Mo and Tl were highest in flathead grey mullet, which was the only marine species. At the same time, the lowest concentrations of As, Cu, Fe, K, Mg, Mn, Mo and Sr were found in eels, and the lowest concentrations of Hg, Se and Mn were found in flathead grey mullet, while the lowest Se:Hg molar ratio was recorded in Prussian carp (Table 4). In general, carnivorous species tend to have higher element levels than planktivorous or herbivorous species [38,39,42,43,47,49,50], although bottom-dwelling species, especially those that dig through sediment and potentially ingest it, may have similar or even higher levels of certain elements than pelagic and benthopelagic species [38,43,47]. That is why, based on the results of several studies [45,51,52,53,54,55], we expected that the eel, as a predatory species with a feeding and habitat ecology primarily in the benthic zone, would contain higher concentrations of a number of elements in its muscle tissues. However, in this study, eels had comparatively higher levels only of Cd, Cr, Na and Se in their muscle, while at the same time the concentrations of As, Cu, Fe, K, Mg, Mn, Mo and Sr were the lowest compared to other analyzed species. Our results are in good agreement with results of Has-Schon et al. [56] and Bukvić et al. [57] for the Neretva River and Rakočević et al. [45] for Skadar Lake, in which eels often had lower concentrations of some elements in comparison to other species. Generally, our results showed that no species had the highest concentrations of all metals (Table 4), indicating that trophic levels and feeding location were not good predictors of element levels, probably because the trophic levels of the analyzed species in this study varied within a relatively narrow range from 2.47 (*P. carp*) to 3.55 (*E. eel*). The highest As values in the flathead grey mullet, which have also been recorded previously [55,56,57], could be expected given that marine fish generally have significantly higher concentrations of organic As species in comparison to freshwater species from unpolluted environments [58]. In addition, IMBI values in the muscles of six fish species (0.130–0.572) also significantly differed between species (Table 4). Hence, IMBI values in muscles of eel, roach and carp were generally higher than those found in other species (eel, roach, carp > barbel, chub, grey mullet), probably due to variations in feeding habits and the behavior of the six species, as suggested by Genç and Yilmaz [59].

It is well known that Se, as an essential trace element vital for metabolic and central nervous system functions [60,61], may counteract MeHg toxicity. It has been suggested that molar excess of Se over Hg, expressed as the Se:Hg molar ratio, protects against MeHg toxicity [62,63,64], where the higher ratios (e.g., 5:1 and higher) are considered to provide better protection against MeHg toxicity than lower ratios [62,65,66,67]. In this study, 94% of samples had Se:Hg molar ratios greater than 5:1, reaching up to 25:1 in the muscle of freshwater species (Italian chub) and 37:1 in flathead grey mullet (Table 4). Higher Se:Hg molar ratios in flathead grey mullet, as the only marine species, could be expected since it is known that freshwater fish species have substantially lower Se levels than marine species [68,69,70,71]. Ratios lower than 5:1 were recorded only in one specimen of Prussian carp and one specimen of Italian barbel, mostly due to an increase in Hg concentrations in those individuals, but even these ratios were significantly higher than 3:1. Accordingly, we can safely conclude that the Se:Hg molar ratios obtained in this study indicate that there was substantial excess of Se over Hg in the muscle tissue of the analyzed species to offer sufficient protection against Hg toxicity.

Since eels are one of two species caught at both stations, and since only the number of eels met the requirements of the analysis of spatial differences, we compared data on the element content in eel muscle tissue between the two locations. Given the spatial differences in the content of elements in the sediments at the observed locations (Table 3), we expected that there might be some locational differences in the eel muscle tissue, with levels of coal-derived elements being higher at the downstream location. However, we found few significant differences in element levels between the locations, and those that we did find were not great. Thus, the concentrations of Cu, Fe, V and Tl were higher at downstream location S2, which could be linked to the potential impact of historical coal mining industry on the lower reaches of the Raša River, while at the same time, concentrations of Hg were higher at location S1 (Table 5). The obtained differences were consistent with the distribution of these elements in the sediment (Table 3). Mass fractions of the remaining elements and Se:Hg molar ratio did not differ between locations although mass fractions of the same elements in sediments differed between sites (Table 3), and this lack of difference probably indicates that the aquatic system of the Raša River is not exposed to significantly elevated concentrations of most of the analyzed elements. In addition, the lack of differences may also be due to the selection of muscle tissue as a biological indicator of contamination, considering that muscle tissue reacts much more slowly to changes in metal content in the environment [72] and that it does not generally serve as a place of accumulation and storage of metals, except for mercury [42,73,74,75,76]. Therefore, we believe that this research should be extended to a larger number of individuals (to increase the power of statistical tests) and to other tissues, such as the liver, which is standardly used to assess the state of metal(loid)s pollution in the organism due to its efficiency of accumulation of most elements, except Hg [77,78,79].

Regardless of the differences between species and indications of rare spatial differences, the overall element levels in the muscle tissues of six fish species from the Raša River were comparable to or lower than the reported data for low-contaminated freshwater systems [9,19,27,43,44,45,51,52,53,54,55,56,57,74,76,80,81,82,83,84,85,86,87,88,89,90,91,92,93,94,95,96,97,98,99,100,101,102,103,104,105,106,107,108,109,110,111,112,113,114,115,116,117,118,119,120,121,122,123] (Tables S12 and S13) and well below the range of the European regulatory limits for Cd (0.05 mg kg^−1^ ww) [124], Hg (0.5 mg kg^−1^ ww) [125] and Pb (0.3 mg kg^−1^ ww) [126], with the exception of Hg in one sample of Prussian carp (0.639 mg kg^−1^ ww). Regarding the Environmental Quality Standards (EQS) for biota [29], which, of all metal(loid)s, were developed only for mercury and its compounds, almost all of our results for THg (which ranged from 20 µg kg^−1^ ww in flathead grey mullet to 639 µg kg^−1^ ww in Prussian carp) exceeded the EQS for THg in biota by up to 32 times (20 µg kg^−1^ ww). However, we believe that there is no cause for concern since the values exceeding the EQS for THg are commonly reported in the literature for European freshwater [46,47,53,84,85,86,87,89,127] and marine fish [49,109] (Table S12), indicating that the established EQS_THg_ value is probably not suitable for biota contamination assessment and should be revised. Our opinion is supported by the fact that the concentrations of THg in the water of the upper and middle reaches of the Raša River were lower than the Canadian Water Quality Guidelines (CWQG) for the protection of aquatic wildlife (0.026 µg L^−1^), while values in sediment were significantly lower than the probable effect concentrations (PEC) for freshwater sediments (0.486 mg kg^−1^ dw), a concentration below which adverse effects are not likely to be observed, indicating that water and sediments from the Raša River are not seriously contaminated with mercury. Overall, our results point to the generally good status of the aquatic ecosystem with regard to the analyzed inorganic elements.

## 4. Conclusions

In this paper, we report for the first time data on a suite of macro and trace elements in water, sediments and six fish species from the upper and middle course of the Raša River (e.g., the western part of the Raša River drainage area) with the intention of assessing the impact of the analyzed elements on living organisms in connection to long-lasting mining activities and the related transport and coal processing industries in the surrounding area.

Levels of the analyzed inorganic elements in water at both locations were low and in accordance with the average values for Croatian stream waters. Compared to international recommendations and regulations, concentrations of total acid leachable elements were significantly lower than the limit values for European inland surface waters and water quality guidelines for the protection of aquatic wildlife. In sediments, most of the elements increased in a downstream direction, which is consistent with the leaching of soils and source rocks in the background and downstream transport and sedimentation of fine-grained material. Regardless of spatial differences, sediments from the upper and middle course of the river contained low to moderate concentrations of metal(loid)s, in accordance with the background composition of the surrounding flysch-derived soils. Although comparison with sediment quality guidelines (SQG) for the protection of aquatic life indicated that existing sediment quality conditions, especially with regard to Cr and Ni, could pose a threat to benthic organisms, the results of bivariate linear regression analysis and factor analysis, together with vertical profiles of elements, sediment quality indices (EF, *Igeo*, PLI) and ecological risk index (RI), showed that the analyzed sediments at both locations mostly reflected the geological and hydrogeological background of the Raša River drainage area, and that the ecological risk of sediment at the investigated locations for benthic organisms was low. In conclusion, levels of most macro and trace elements in water and sediments were low, indicating that the aquatic system of the upper and middle reaches of the Raša River was not significantly loaded with toxic elements.

This was also reflected in the overall element levels in the muscle tissues of fish species from the Raša River, which were in accordance with the values typical for low-contaminated freshwater systems and mostly well below the European regulatory limits for toxic elements in foodstuffs. The holistic approach which was used in this study, which included analyses of a large number of elements in water, sediments and fish in the investigated area, together with the application of statistical methods, enabled us to comprehensively investigate the behavior of inorganic elements in the upper and middle course of the Raša River. Based on the results of this analysis, we showed that, unlike the eastern part of the Raša River drainage area and the estuary, the western part of the basin was not significantly polluted with regard to the analyzed inorganic elements.

## Figures and Tables

**Table 1:** Basic parameters of the six fish species collected at two locations in the Raša River. Data for fork length, body weight and water content are presented as median (range).

Name	Feeding Habit	Trophic Level ^3^	Sampling Location	Number of Individuals /Composite Samples	Fork Length (cm)	Body Weight (g)	Water Content (%)
European eel ^1^; *Anguilla anguilla* (Linnaeus, 1758)	mainly carnivore (fish, mollusks, polychaetes, insects, crustaceans, detritus)	3.55 ± 0.30	S1, S2	6/6	39.1 (34.1–43.1)	122.9 (64.3–161.9)	55.2 (49.6–72.8)
Prussian carp ^2^; *Carassius gibelio* (Bloch, 1782)	plankton, benthic invertebrates, plant material, detritus	2.47 ± 0.04	S1	2/-	26.6 (26.2–26.9)	446.7 (418.5–475.0)	75.7 (74.6–76.9)
Flathead grey mullet ^1^; *Mugil cephalus* (Linnaeus, 1758)	zooplankton, dead plant matter, detritus, epiphytes and epifauna from algae, sediment	2.48 ± 0.17	S2	6/-	29.2 (25.6–33.0)	235.2 (177.5–325.4)	78.4 (73.5–81.2)
Italian barbel ^2^; *Barbus plebejus* Bonaparte, 1839	benthic invertebrates, small fish and algae	3.38 ± 0.51	S1	4/2	17.5 (11.4–23.5)	62.5 (16.0–206.4)	79.1 (75.1–80.0)
Adriatic roach ^2^; *Rutilus aula* (Bonaparte, 1841)	detritus, algae, planktonic organisms, smaller aquatic invertebrates	2.80 ± 0.30	S1	-/4	9.6 (9.1–11.8)	12.9 (8.6–18.5)	77.5 (77.3–79.52)
Italian chub ^2^; *Squalius squalus* (Bonaparte, 1837)	various aquatic and terrestrial animal and plant material	3.40 ± 0.50	S1, S2	3/5	19.0 (8.4–27.9)	105.4 (8.1–317.0)	77.4 (74.9–89.7)

^1^ euryhaline species; ^2^ freshwater species; ^3^ Trophic level data were obtained from FishBase (www.fishbase.org; accessed on 15 September 2022).

**Table 2:** Comparison of average levels (median (range)) of elements (in µg/L unless otherwise stated) in unfiltered water samples collected in June and July of 2020 at 2 locations in the Raša River with the relevant published data (^1–3^) and water quality guidelines (^4,5^).

	Present Study		Published Data					
	S1	S2	Raša River Estuary ^1^	Raša River Estuary ^2^	Natural Freshw. Spring ^2^	Stream Water; Average for Croatia ^3^	MAC ^4^	CWQG ^5^
Ca (mg/L)	95.5 (95.3–95.8)	101 (100–102)	-	-	-	94.6 (21.2–120)	-	-
K (mg/L)	1.48 (1.44–1.51)	1.54 (1.52–1.55)	-	-	-	2.00 (0.85–4.68)	-	-
Mg (mg/L)	9.57 (9.55–9.59)	6.18 (6.15–6.21)	-	-	-	28.6 (7.3–75.2)	-	-
Na (mg/L)	10.7 (10.7–10.8)	8.38 (8.28–8.47)	-	-	-	11.9 (4.4–37.1)	-	-
Ag	0.003 (0.001–0.004)	0.003 (0.0031–0.0032)	-	-	-	0.001 (0.001–0.001)	-	0.25
As	0.503 (0.497–0.508)	0.303 (0.297–0.309)	0.51–0.61	-	0.40–1.40	2.30 (0.95–22.1)	-	5.0
Ba	43.8 (43.5–44.2)	35.6 (35.2–36.0)	16.5–40.2	31.3	14.6	36.7 (14.4–63.3)	-	-
Cd	0.021 (0.019–0.023)	0.011 (0.011–0.012)	<LOD	0.21	0.03–0.2	0.0035 (0.001–0.029)	0.9	0.09
Co	0.143 (0.141–0.144)	0.095 (0.095–0.096)	0.03–0.05	0.06	0.04	0.20 (0.15–1.11)	-	-
Cr	0.669 (0.668–0.670)	0.615 (0.611–0.618)	0.26–0.40	0.60	0.5–5	0.79 (0.29–4.81)	-	-
Cs	0.015 (0.013–0.018)	0.007 (0.007–0.008)	0.06–0.10	0.04	0.004	0.001 (0.001–0.012)	-	-
Cu	1.16 (1.11–1.20)	0.732 (0.707–0.756)	0.33–1.81	0.60	0.43–10	0.935 (0.720–2.13)	-	2.0
Fe	231 (227–234)	64.1 (60.4–67.8)	12.6–33.0	2.1	1–390	27.8 (4.80–911)	-	300
Hg	0.012 (0.011–0.012)	0.013 (0.011–0.015)	-	-	-	-	0.07	0.026
Mn	50.0 (49.6–50.4)	7.08 (7.04–7.12)	8.68–9.49	2.2	0.5–25	66.1 (0.500–668)	-	-
Mo	1.57 (1.53–1.62)	1.69 (1.68–1.70)	3.91–6.06	33.1	2.04	0.400 (0.100–1.10)	-	73
Ni	1.12 (1.08–1.15)	0.723 (0.710–0.736)	<LOD	1.3	0.35–20	2.45 (1.99–4.94)	34	25
Pb	0.317 (0.312–0.321)	0.096 (0.091–0.101)	0.39–2.12	0.2	0.09–1	0.062 (0.044–0.240)	14	1.0
Rb	1.11 (1.08–1.14)	0.865 (0.865–0.865)	28–7-51.0	19.5	1.04	0.620 (0.220–2.18)	-	-
Sb	0.045 (0.043–0.047)	0.035 (0.034–0.037)	0.07–0.09	0.37	0.07	0.120 (0.040–0.160)	-	-
Se	0.207 (0.194–0.219)	0.228 (0.217–0.239)	0.34–0.48	3.50	1.09	0.205 (0.099–0.77)	-	1.0
Sn	0.049 (0.047–0.051)	0.029 (0.028–0.030)	0.12–0.34	0.35	0.35	-	-	-
Sr	336 (331–340)	621 (613–630)	2316–3691	1797	191	227 (69–493)	-	-
Tl	0.004 (0.004–0.005)	0.007 (0.007–0.008)	0.012–0.014	0.04	0.01	0.002 (0.001–0.006)	-	0.8
U	0.528 (0.525–0.530)	0.621 (0.621–0.622)	1.27–1.77	10.8	0.75	1.30 (0.044–4.13)	-	15
V	0.770 (0.746–0.793)	0.787 (0.774–0.799)	0.77–1.05	1.70	1.37	1.15 (0.290–1.74)	-	-
Zn	6.55 (6.49–6.60)	2.82 (2.60–3.42)	2.17–5.04	4.20	2.5–267	1.02 (0.100–1.58)	-	7.0

<LOD—below the detection limit of the method; ^1^ [14]; ^2^ [7]; ^3^ [28]; ^4^ maximum allowable concentrations (MAC) for inland surface waters [29]; ^5^ Canadian water quality guidelines (CWWGs) for the protection of aquatic wildlife [30]—values are for the long-term exposure.

**Table 3:** Average concentrations (arithmetic mean ± SD [range]) of inorganic elements in sediments from the Raša River (in mg kg^−1^ dry weight except when stated otherwise) compared with relevant published data (^1–5^) and sediment quality guidelines (^6–8^).

	Present Study		Published Data							
	S1 (N = 9)	S2 (N = 6)	Raša River ^1^	Istrian Rivers ^2^	Raša Riv. Estuary ^3^	Istrian Flysch Soils ^4^	World av., Stream Sedim. ^5^	ISQG ^6^	PEL ^7^	TEC ^8^
Al (%)	2.73 ± 0.13 ^a^ (2.50–2.87)	4.25 ± 0.11 ^b^ (4.07–4.37)	0.99–1.11	1.32 ± 0.44	3.43–4.38	5.27 ± 1.05	5.22 (0.78–8.51)	-	-	-
Ca (%)	13.70 ± 0.41 ^a^ (13.2–14.4)	8.53 ± 0.27 ^b^ (8.10–8.86)	10.5–10.8	10.8 ± 7.3	-	9.35 ± 6.15	0.486 (<0.048–9.98)	-	-	-
Fe (%)	2.43 ± 0.25 ^a^ (2.05–2.80)	3.62 ± 0.04 ^b^ (3.58–3.68)	1.77–1.90	2.66 ± 0.90	1.90–2.47	2.68 ± 0.34	2.5 (0.678–13.7)	-	-	2
K (%)	0.948 ± 0.062 ^a^ (0.813–1.02)	1.40 ± 0.01 ^b^ (1.38–1.42)	0.10–0.14	0.15 ± 0.06	-	1.54 ± 0.13	1.63 (0.266–3.49)	-	-	-
Mg (%)	0.655 ± 0.021 ^a^ (0.626–0.687)	0.806 ± 0.009 ^b^ (0.790–0.816)	0.43–0.51	0.57 ± 0.17	-	0.69 ± 0.08	0.458 (0.066–3.27)	-	-	-
Na (%)	0.367 ± 0.023 ^a^ (0.321–0.388)	0.332 ± 0.006 ^b^ (0.324–0.342)	-	0.03 ± 0.05	-	0.552 ± 0.124	0.683 (0.045–1.34)	-	-	-
Ag	0.179 ± 0.012 ^a^ (0.159–0.200)	0.220 ± 0.007 ^b^ (0.215–0.233)	-	-	-	<DL	0.09 (0.022–0.31)	-	-	1.0
As	5.88 ± 0.86 ^a^ (4.74–7.25)	7.52 ± 0.60 ^b^ (6.72–8.21)	3.3–4.8	6.54 ± 2.47	6.12–6.52	7.77 ± 2.12	2.9 (0.53–23.8)	5.9	17	9.8
B	66.3 ± 2.3 ^a^ (62.7–69.8)	76.9 ± 1.0 ^b^ (75.4–78.4)	-	7.69 ± 6.78	-	-	23 (<1.0–110)	-	-	-
Ba	220 ± 10 ^a^ (202–235)	270 ± 6 ^b^ (261–277)	55–120	116 ± 52	148–168	218 ± 38	376 (127–5686)	-	-	-
Cd	0.288 ± 0.025 ^a^ (0.247–0.326)	0.474 ± 0.032 ^b^ (0.437–0.518)	0.2–0.3	0.26 ± 0.13	0.29–0.44	<DL	1.8 (0.09–61)	0.6	3.5	0.99
Co	14.0 ± 1.5 ^a^ (11.6–16.0)	16.8 ± 0.5 ^b^ (16.3–17.4)	10.2–11.1	17.9 ± 8.43	11.1–13.8	13.7 ± 2.8	17 (<7–220)	-	-	50
Cr	140 ± 13 (127–165)	149 ± 3 (144–155)	30.6–31.3	44.9 ± 16.4	106–125	95.3 ± 13.4	69 (20–448)	37.3	90	43.4
Cs	8.27 ± 0.49 ^a^ (7.51–8.85)	13.6 ± 0.38 ^b^ (13.1–14.1)	-	0.98 ± 0.50	3.89–5.74	-	8.2 (3.3–52)	-	-	-
Cu	26.3 ± 0.5 ^a^ (25.8–27.6)	37.4 ± 2.6 ^b^ (35.8–41.8)	21.7–24.6	28.9 ± 9.61	22.0–32.0	30.8 ± 4.36	23 (<10–2440)	35.7	197	31.6
Hg	0.152 ± 0.022 (0.123–0.185)	0.179 ± 0.036 (0.133–0.229)	0.115–0.358	2.49 ± 10.3	-	0.038 ± 0.012	0.09 (<0.01–3.3)	0.170	0.486	0.180
Li	38.7 ± 1.7 ^a^ (36.5–40.6)	61.9 ± 1.5 ^b^ (60.0–63.4)	-	23.0 ± 8.58	34.7–46.2	-	31 (2–90)	-	-	-
Mn	758 ± 80 ^a^ (599–895)	653 ± 56 ^b^ (580–733)	563–732	969 ± 426	504–511	720 ± 137	1394 (77–51,743)	-	-	460
Mo	2.01 ± 0.70 ^a^ (0.948–3.26)	0.593 ± 0.024 ^b^ (0.566–0.630)	-	-	0.79–2.53	-	1.5 (0.19–8.5)	-	-	-
Ni	73.2 ± 4.20 ^a^ (65.9–79.1)	98.8 ± 2.6 ^b^ (95.8–102)	51.0–61.9	85.6 ± 32.4	53.6–72.3	73.1 ± 11.2	28 (<7–508)	-	-	22.7
P	400 ± 18 ^a^ (369–419)	606 ± 20 ^b^ (572–624)	-	380 ± 200	-	410 ± 110	655 (175–3753)	-	-	-
Pb	14.0 ± 1.1 ^a^ (12.8–16.5)	16.6 ± 1.0 ^b^ (15.4–17.9)	12.2–14.5	21.2 ± 12.9	17.7–18.5	18.1 ± 4.04	195 (24–9870)	35	91.3	35.8
Sb	0.613 ± 0.153 (0.521–0.977)	0.603 ± 0.045 (0.553–0.677)	-	0.27 ± 0.15	0.47–0.91	<DL	2.7 (0.8–10)	-	-	2
Se	0.875 ± 0.567 (0.372–2.17)	0.627 ± 0.097 (0.545–0.795)	-	0.63 ± 0.45	0.34–0.48	-	2.3 (0.1–8.6)	-	-	2
Sn	1.31 ± 0.12 ^a^ (1.18–1.50)	2.44 ± 0.47 ^b^ (2.06–3.25)	-	1.11 ± 1.25	0.12–0.34	<DL	6 (<3–57)	-	-	-
Sr	361 ± 9 ^a^ (345–374)	256 ± 2 ^b^ (253–260)	-	225 ± 124	312–317	199 ± 100	125 (4–498)	-	-	-
Tl	0.347 ± 0.015 ^a^ (0.324–0.370)	0.658 ± 0.027 ^b^ (0.615–0.691)	-	0.15 ± 0.06	0.33–0.46	-	0.79 (0.2–2.5)	-	-	-
U	1.75 ± 0.22 ^a^ (1.60–2.26)	2.15 ± 0.06 ^b^ (2.05–2.22)	-	0.49 ± 0.20	1.97–2.91	-	6.5 (2–71)	-	-	-
V	77.9 ± 2.7 ^a^ (73.0–81.6)	121 ± 3.2 ^b^ (117–125)	-	32.8 ± 9.39	74.2–101	94.6 ± 13.0	58 (17–151)	-	-	-
Zn	62.4 ± 1.5 ^a^ (59.5–63.8)	97.5 ± 2.8 ^b^ (92.9–101)	50.3–58.6	70.4 ± 26.5	66.2–95.8	67.6 ± 1.9	45.9 (14.2–165)	123	315	121

^1^ Average for Raša River sediments; elements determined in sediment fraction <63 µm [14]; ^2^ average for Istrian river sediments; elements determined in sediment fraction <63 µm [14]; ^3^ Raša River Estuary [14]; ^4^ background values for Istrian Flysch-derived soils [20]; ^5^ world average for stream sediment, fraction <63 µm [31]; ^6^ ISQG—Interim Freshwater Sediment Quality Guidelines [33]; ^7^ PEL—probable effect level [33]; ^8^ TEC—threshold effect concentration [34]. Differences between locations were tested with nonparametric Mann–Whitney U test. Different superscript letters (^ab^) within the row indicate significant differences in element levels between sampling locations.

**Table 4:** Macro and trace element concentrations (in mg kg^−1^ or µg kg^−1^ wet weight) and median (range) Se:Hg molar ratios and IMBI values in the muscle tissue of six fish species from the Raša River, Istria, Croatia.

	European Eel (N = 12)	Prussian Carp (N = 2)	Italian Barbel (N = 6)	Adriatic Roach (N = 4)	Italian Chub (N = 8)	Flathead Grey Mullet (N = 6)
Ca (mg kg^−1^)	223 (153–286) ^a,c^	348 (223–472) ^a,b,c^	447 (397–683) ^c^	3944 (531–4837) ^c^	485 (171–3855) ^c^	137 (92–316) ^b^
K (mg kg^−1^)	2228 (1846–2887) ^a^	2744 (2562–2925) ^a,b^	2690 (2562–3338) ^a,b^	2639 (2481–2837) ^a,b^	3234 (1236–3539) ^b^	3524 (3248–3727) ^b^
Na (mg kg^−1^)	504 (435–669) ^a^	306 (276–337) ^b^	425 (309–483) ^a,b^	425 (411–491) ^a,b^	377 (183–403) ^b^	351 (268–367) ^b^
Mg (mg kg^−1^)	186 (140–237) ^a^	258 (236–280) ^a,b^	265 (246–280) ^b^	306 (250–325) ^b^	250 (148–337) ^b^	244 (203–256) ^a,b^
Ag (µg kg^−1^)	0.524 (0.084–1.42)	0.230 (0.202–0.258)	0.160 (0.082–1.95)	0.357 (0.123–1.26)	0.204 (0.098–0.916)	0.172 (0.097–0.370)
As (mg kg^−1^)	0.047 (0.023–0.078) ^a^	0.045 (0.042–0.049) ^a,b^	0.061 (0.020–0.119) ^a,b^	0.066 (0.058–0.077) ^a,b^	0.031 (0.020–0.057) ^b^	0.422 (0.364–0.709) ^c^
Cd (µg kg^−1^)	2.17 (1.19–3.25) ^a^	0.146 (0.145–0.148) ^b^	0.379 (0.195–0.581) ^b^	1.00 (0.68–1.23) ^a,b^	0.857 (0.374–1.33) ^a,b^	0.312 (0.252–0.367) ^b^
Co (mg kg^−1^)	0.045 (0.017–0.121) ^a^	4.51 (0.060–8.96) ^b^	0.036 (0.006–0.108) ^a^	0.072 (0.022–0.153) ^a^	0.035 (0.003–0.713) ^a^	0.013 (0.009–0.026) ^a^
Cr (mg kg^−1^)	0.016 (0.004–0.031) ^a^	0.012 (0.002–0.021) ^a,b^	0.001 (0.0002–0.009) ^b^	0.004 (0.002–0.008) ^a,b^	0.003 (0.001–0.017) ^b^	0.004 (0.003–0.009) ^a,b^
Cu (mg kg^−1^)	0.214 (0.157–0.319) ^a^	0.307 (0.219–0.396) ^a,b^	0.307 (0.187–0.444) ^a,b^	0.253 (0.208–0.392) ^a,b^	0.341 (0.171–0.417) ^a,b^	0.373 (0.264–0.670) ^b^
Fe (mg kg^−1^)	3.01 (2.01–4.60) ^a,c^	5.58 (4.49–6.67) ^a,b,c^	2.62 (2.22–3.96) ^c^	3.79 (2.83–5.18) ^a,b,c^	3.36 (1.96–4.84) ^c^	8.16 (5.17–10.2) ^b^
Hg (mg kg^−1^)	0.205 (0.158–0.329) ^a,c^	0.420 (0.201–0.639) ^c^	0.141 (0.097–0.238) ^a,b,c^	0.108 (0.081–0.135) ^a,b,c^	0.079 (0.043–0.194) ^b^	0.036 (0.020–0.060) ^b^
Mn (mg kg^−1^)	0.239 (0.159–0.302) ^a,c^	0.272 (0.179–0.365) ^a,b^	0.489 (0.416–0.625) ^b,c^	2.41 (0.49–4.06) ^b^	0.514 (0.214–2.17) ^b,c^	0.166 (0.122–0.372) ^a^
Mo (µg kg^−1^)	1.76 (0.72–3.18) ^a^	2.40 (2.12–2.68) ^a,b^	2.75 (1.47–4.09) ^a,b^	9.57 (6.44–10.6) ^b^	4.77 (2.90–7.45) ^b^	29.8 (2.91–89.4) ^b^
Ni (µg kg^−1^)	16.2 (9.3–37.6) ^a^	29.8 (5.60–54.1) ^a,b^	7.12 (3.12–9.62) ^b^	21.9 (10.7–30.1) ^a^	9.08 (1.94–23.6) ^a,b^	8.22 (5.31–9.48) ^a,b^
Pb (µg kg^−1^)	2.88 (1.04–18.0)	4.65 (3.61–5.70)	1.80 (0.45–2.11)	11.3 (1.21–14.8)	1.22 (0.63–3.59)	3.54 (0.85–5.06)
Se (mg kg^−1^)	1.33 (0.857–1.54) ^a^	0.909 (0.880–0.937) ^a,b^	0.702 (0.417–1.22) ^a,b^	0.739 (0.690–0.795) ^a,b^	0.601 (0.365–0.803) ^b^	0.265 (0.226–0.587) ^b^
Sr (mg kg^−1^)	0.309 (0.193–0.562) ^a^	0.450 (0.297–0.603) ^a,b^	0.657 (0.591–1.16) ^a,b^	7.59 (0.97–8.97) ^b^	0.810 (0.341–4.80) ^b^	0.501 (0.216–1.76) ^a^
Tl (µg kg^−1^)	2.16 (0.81–3.52) ^a^	0.390 (0.242–0.538) ^a,b^	1.10 (0.44–2.03) ^a,b^	1.03 (0.51–1.46) ^a,b^	0.800 (0.381–1.47) ^b^	4.22 (0.43–8.93) ^a,b^
U (µg kg^−1^)	0.104 (0.067–0.165) ^a,b^	0.035 (0.028–0.042) ^a^	0.047 (0.030–0.060) ^a^	0.362 (0.085–0.536) ^b^	0.082 (0.026–0.263) ^a,b^	0.097 (0.054–0.177) ^a,b^
V (µg kg^−1^)	2.04 (1.37–5.01)	1.60 (1.31–1.88)	3.21 (2.07–5.56)	8.87 (2.12–12.3)	3.20 (1.13–7.59)	4.50 (2.87–9.46)
Zn (mg kg^−1^)	18.2 (14.0–27.7) ^a^	7.88 (6.68–9.09) ^b^	5.68 (4.40–6.85) ^b^	21.9 (11.9–25.7) ^a^	11.0 (6.78–20.0) ^a,b^	7.78 (7.10–10.0) ^b^
Se:Hg molar ratio	16.2 (11.0–22.9) ^a^	7.42 (3.73–11.1) ^b^	14.4 (4.45–24.3) ^a^	18.1 (14.1–21.6) ^a^	21.0 (10.5–24.6) ^a^	25.1 (10.8–36.9) ^a^
IMBI	0.424 (0.295–0.572) ^a^	0.376 (0.237–0.516) ^a,b^	0.182 (0.138–0.256) ^b^	0.411 (0.212–0.504) ^a,b^	0.229 (0.130–0.401) ^a,b^	0.216 (0.176–0.286) ^b^

IMBI—Individual Mean Bioaccumulation Index based on 7 metals (Cd, Cr, Cu, Hg, Ni, Pb, Zn) [26]. Different superscript letters (^abc^) within the row indicate differences in element levels between species. Differences were tested with nonparametric using Kruskall–Wallis H test. Means with the same letter are not significantly different across sites.

**Table 5:** Biometric parameters, water content (%) and average concentrations of elements (in mg kg^−1^ or µg kg^−1^ wet weight), Se:Hg molar ratios and IMBI [median (range)] in the muscle tissue of the European eel from the Raša River grouped by location.

	S1 (N = 6)	S2 (N = 6)	*p*-Value
TL (cm)	40.3 (34.3–42.9)	38.8 (34.1–43.1)	n.s.
TBW (g)	111 (64–148)	136 (77–162)	n.s.
Water content (%)	55.1 (50.7–62.1)	55.2 (49.6–72.8)	n.s.
Ca (mg kg^−1^)	214 (184–229)	227 (153–286)	n.s.
K (mg kg^−1^)	2228 (1863–2528)	2263 (1846–2887)	n.s.
Mg (mg kg^−1^)	180 (140–196)	187 (148–237)	n.s.
Na (mg kg^−1^)	504 (435–587)	505 (435–669)	n.s.
Ag (µg kg^−1^)	0.457 (0.084–0.651)	0.737 (0.384–1.416)	n.s.
As (mg kg^−1^)	0.044 (0.029–0.078)	0.049 (0.023–0.069)	n.s.
Cd (µg kg^−1^)	1.39 (1.19–3.25)	2.28 (1.29–3.24)	n.s.
Co (mg kg^−1^)	0.046 (0.017–0.105)	0.045 (0.030–0.121)	n.s.
Cr (mg kg^−1^)	0.011 (0.005–0.016)	0.018 (0.004–0.031)	n.s.
Cu (mg kg^−1^)	0.178 (0.157–0.245)	0.232 (0.209–0.319)	0.300
Fe (mg kg^−1^)	2.44 (2.01–3.44)	3.30 (2.68–4.60)	0.045
Hg (mg kg^−1^)	0.235 (0.186–0.329)	0.170 (0.158–0.224)	0.031
Mn (mg kg^−1^)	0.238 (0.159–0.295)	0.252 (0.194–0.302)	n.s.
Mo (µg kg^−1^)	1.47 (0.72–2.80)	2.13 (1.14–3.18)	n.s.
Ni (µg kg^−1^)	14.2 (12.0–18.9)	20.4 (9.3–37.6)	n.s.
Pb (µg kg^−1^)	4.0 (1.8–18.0)	2.32 (1.04–5.12)	n.s.
Se (mg kg^−1^)	1.36 (1.11–1.43)	1.25 (0.86–1.54)	n.s.
Sr (mg kg^−1^)	0.305 (0.234–0.562)	0.312 (0.193–0.446)	n.s.
Tl (µg kg^−1^)	1.73 (0.81–3.20)	2.52 (2.05–3.52)	0.044
U (µg kg^−1^)	0.108 (0.092–0.165)	0.100 (0.067–0.157)	n.s.
V (µg kg^−1^)	1.94 (1.37–2.13)	3.47 (1.81–5.01)	0.031
Zn (mg kg^−1^)	17.7 (14.0–22.5)	18.5 (14.8–27.7)	n.s.
Se:Hg molar ratio	13.4 (11.0–18.9)	17.1 (12.8–22.9)	n.s.
IMBI*	0.412 (0.302–0.521)	0.431 (0.378–0.591)	n.s.

TL—total fork length (cm); TBW—total body weight (g); IMBI*—Individual Mean Bioaccumulation Index based on 7 metals (Cd, Cr, Cu, Hg, Ni, Pb, Zn) [27]; Differences between locations were tested with nonparametric Mann–Whitney U test and considered significant at *p* < 0.05.

## Data Availability

The data presented in this study are available on request from the corresponding author. The data are not publicly available due to privacy issues.

## Supplementary Materials

The following supporting information can be downloaded at: https://www.mdpi.com/article/10.3390/toxics11010042/s1, Table S1: ICP-MS 7500cx (Agilent Technologies, Tokyo, Japan) optimized working conditions; Table S2: Limits of detection (LOD) of two methods for determination of major and trace elements by ICP-MS in waters (in µg L^−1^), fish tissue (in mg kg^−1^ wet mass (wm)) and sediment (in mg kg^−1^ dry matter (dm)) collected in Raša River in June of 2020; Table S3: Temperature program for digestion of fish tissues and sediment in the microwave digestion system UltraCLAVE IV (Milestone Srl, Sorisole, Italy); Table S4: Grades on the basis of *EF, I*_geo_, and PLI values; Table S5: Grade standards for *E_r_^i^* and RI; Table S6: Correlation coefficients for mass fractions of 30 elements in sediments of the upper and middle course of the Raša River. Marked correlations are significant at *p* < 0.05 (N = 15); Table S7: Results of the principal component analysis (PCA) (Varimax rotated) of concentration of elements in sediments from the Raša River. Significant correlations (r > 0.7) are shown in bold; Table S8: The enrichment factors (EF) of metal(loid)s in the sediments; Table S9: Index of geo-accumulation (*Igeo*) of metal(loid)s in the sediments; Table S10: Pollution index (PLI) of metal(loid)s in the sediments; Table S11: *E_r_^i^* and RI of metal(loid)s in the sediments; Table S12: Comparison of average concentrations [arithmetic mean (range) or arithmetic mean ± standard deviation] of potentially toxic elements [in mg kg^−1^ or µg kg^−1^ wet mass (wm)] in muscle tissue of six freshwater fish species from the Raša River (Istria, Croatia) with the literature data for similar freshwater species from European rivers, lakes and reservoirs; Table S13: Comparison of average concentrations [arithmetic mean (range) or arithmetic mean ± standard deviation] of macro (Ca, K, Mg, Na) and trace (Ag, Fe, Mo, Sr, Tl, V) elements [in mg kg^−1^ or µg kg^−1^ wet mass (wm)] in muscle tissue of six freshwater fish species from the Raša River (Istria, Croatia) with the literature data for similar freshwater species from European rivers, lakes and reservoirs.
