# Supplementary material for: Potentially Toxic Elements in Water, Sediments and Fish from the Karstic River (Raša River, Croatia) Located in the Former Coal-Mining Area
Source: Toxics. 2022 Dec 31;11(1):42. doi: 10.3390/toxics11010042 (PMC9865867; doi:10.3390/toxics11010042)
Supplement: Supplementary file 1 [file toxics-11-00042-s001.zip › toxics-2119646-supplementary.pdf]

Toxics

Supplementary Materials for

Potentially toxic elements (PTEs) in water, fish and sediments from the karstic river (Raša River, Croatia) located in the abandoned coal-mining area

By: Zorana KLJAKOVIĆ-GAŠPIĆ*, Ankica SEKOVANIĆ, Tatjana ORCT, Dora ŠEBEŠĆEN, Elena KLASIČEK, Davor ZANELLA

Corresponding author:
*Zorana Kljaković-Gašpić
Institute for Medical Research and Occupational Health (IMROH)
Ksaverska cesta 2, HR−10000 Zagreb, Croatia
e-mail: zorana@imi.hr
Phone: 00385 1 4682 534
Fax: 00385 1 4673 303

Content

1	ICP-MS 7500cx (Agilent Technologies, Japan) optimized working conditions.	Table S1	Page 2
2	Limits of detection (LOD) of the methods for determination of major and trace elements by ICP-MS in water, fish tissue and sediment	Table S2	Page 3
3	Temperature program for digestion of fish tissues and sediment in the microwave digestion system UltraCLAVE IV (Milestone, Sorisole, Italy)	Table S3	Page 4
4	Grades on the basis of *EF*, I_{geo}, and *PLI* values	Table S4	Page 4
5	Grade standards for E_r^i and *RI*	Table S5	Page 4
6	Correlation coefficients for mass fractions of 30 elements in sediments of the upper and middle course of the Raša River	Table S6	Page 5
7	Results of the Principal Components Analysis (PCA) (Varimax rotated) of concentration of elements from the Raša River	Table S7	Page 6
8	The enrichment factors (EF) of metal(loid)s in the sediments	Table S8	Page 7
9	Index of geo-accumulation (I_{geo}) of metal(loid)s in the sediments	Table S9	Page 8
10	Pollution index (PLI) of metal(loid)s in the sediments	Table S10	Page 9
11	E_r^i and *RI* of metal(loid)s in the sediments	Table S11	Page 10
12	Comparison of average concentrations of potentially toxic elements in muscle tissue of six freshwater fish species from the Raša River with the literature data	Table S12	Pages 11–14
13	Comparison of average concentrations of macro and trace elements in muscle tissue of six freshwater fish species from the Raša River with the literature data	Table S13	Pages 15–16

Table S1 ICP-MS 7500cx (Agilent Technologies, Tokyo, Japan) optimized working conditions.

**Parameter**			
RF Power (W)	1550		
RF matching (V)	1.68		
Sampling depth (mm)	7.5		
Torch-H (mm)	0.4		
Torch-V (mm)	−0.4		
Nebulizer pump flow (rps)	0.08		
Plasma gas flow rate (L min^{-1})	15		
Makeup gas flow rate (L min^{-1})	0.1		
Carrier gas flow rate (L min^{-1})	1.05		
Nebulizer	MicroMist (quartz)		
Spray chamber	Scott type (quartz), cooled at 2 °C		
Sample cone	Nickel, 1 mm orifice diameter		
Skimmer cone	Nickel, 0.4 mm orifice diameter		
Doubly-charged ions and oxides limits	$^{140}Ce^{2+}/^{140}Ce^{+} < 2.2\%$ $^{140}Ce^{16}O^{+}/^{140}Ce^{+} < 1.4\%$		
**Collision/reaction gas**	**No gas**	**H_2**	**He**
Collision/reaction gas flow rate (L min^{-1})	/	4.3	4.2
Extract lens 1 voltage (V)	0	0	0.5
Extract lens 2 voltage (V)	−150	−150	−135
Isotopes measured	^{7}Li, ^{11}B, ^{27}Al, ^{202}Hg, ^{238}U	^{78}Se	^{23}Na, ^{24}Mg, ^{34}S, ^{39K}, ^{43}Ca, ^{51}V, ^{53}Cr, ^{55}Mn, ^{56}Fe, ^{59}Ca, ^{60}Ni, ^{63}Cu, ^{68}Zn, ^{75}As, ^{85}Rb, ^{88}Sr, ^{95}Mo, ^{107}Ag, ^{114}Cd, ^{118}Sn, ^{121}Sb, ^{133}Cs, ^{138}Ba, ^{205}Tl, ^{208}Pb

Table S2 Limits of detection (LOD) of two methods for determination of major and trace elements by ICP-MS in waters (in μg L^{-1}), fish tissue (in mg kg^{-1} wet mass (wm)) and sediment (in mg kg^{-1} dry matter (dm)) collected in Raša River in June of 2020.

Element	LOD water [a] (μg L^{-1})	LOD fish (mg kg^{-1} w.m.)	LOD sediment (mg kg^{-1} d.m.)
**Ag**	0.001	0.00003	0.04
**Al**	contamination	not measured	25.6
**As**	0.0015	0.00007	0.6
**B**	not measured	0.002	1.4
**Ba**	0.05	not measured	not measured
**Ca**	1.5	0.00002	0.08
**Cd**	0.0004	0.00003	0.016
**Co**	0.0005	0.00044	0.12
**Cr**	0.003	0.000002	2.35
**Cs**	not measured	not measured	0.004
**Cu**	0.1	0.02	1.86
**Fe**	0.045	0.00044	45.9
**Hg**	0.0015	0.11	0.02
**K**	1.9	0.0002	0.9
**Li**	0.02	0.015	2.6
**Mg**	0.09	0.002	0.023
**Mn**	0.004	0.00015	0.38
**Mo**	0.015	0.23	0.02
**Na**	0.9	0.001	1.16
**Ni**	0.021	0.00004	1.35
**P**	not measured	not measured	0.29
**Pb**	0.005	0.000007	0.16
**Rb**	0.013	not measured	not measured
**Sb**	0.003	not measured	0.064
**Se**	0.0045	0.00004	0.36
**Sn**	0.003	not measured	0.073
**Sr**	0.015	0.000003	0.135
**Tl**	0.015	0.000003	0.0068
**U**	0.0014	0.00002	0.002
**V**	0.001	0.006	0.11
**Zn**	0.1	0.00003	10.7

[a] Detection limit of the analysis was calculated according to the equation: $LOD = X_{blank} + K \times SD_{blank}$, where: X_{blank} was average element concentration in procedural blank sample (six independent determinations of each blank), K = 3, and SD_{blank} was the standard deviation of six independent determinations of all procedural blanks.

Table S3 Temperature program for digestion of fish tissues and sediment in the microwave digestion system UltraCLAVE IV (Milestone Srl, Sorisole, Italy).

	Fish tissues				Sediment			
STEP	Time (min:s)	Energy (W)	Temp. (°C)	Pressure (bar)	Time (min:s)	Energy (W)	Temp. (°C)	Pressure (bar)
1.	3:30	700	70	100	12	200	75	100
2.	15	1000	180	100	25	300	125	100
3.	10	1000	250	140	10:30	500	160	100
4.	30	1000	250	140	12:30	1000	240	160
5.	40	0	30	20	40	1000	240	160
6.	-	-	-	-	30	0	30	20

Table S4 Grades on the basis of *EF*, I_{geo}, and *PLI* values.

EF	**Enrichment level**	I_{geo}	**Pollution level**	*PLI*	**Pollution level**
$EF < 1$	Deficiency	$I_{geo} \leq 0$	Unpolluted	$0 < PLI \leq 1$	Unpolluted
$1 \leq EF < 2$	minimal enrichment	$0 < I_{geo} \leq 1$	Slightly polluted	$1 < PLII \leq 2$	Unpolluted to moderately polluted
$2 \leq EF < 5$	Moderate enrichment	$1 < I_{geo} \leq 2$	Moderately polluted	$2 < PLI \leq 3$	Moderately polluted
$5 \leq EF < 20$	Significant enrichment	$2 < I_{geo} \leq 3$	Moderately to heavily	$3 < PLI \leq 4$	Moderately to highly polluted
$20 \leq EF < 40$	Very high enrichment	$3 < I_{geo} \leq 4$	Heavily polluted	$4 < PLI \leq 5$	Highly polluted
$40 \leq EF$	Extremely high enrichment	$4 < I_{geo} \leq 5$	Heavily to extremely polluted		
		$I_{geo} > 5$	Extremely polluted		

Table S5 Grade standards for E_r^i and *RI.*

E_r^i	**Ecological risk grade**	*RI*	**Ecological risk grade**
$E_r^i < 15$	Low	$RI < 50$	Low
$15 \leq E_r^i < 30$	Moderate	$50 \leq RI < 100$	Moderate
$30 \leq E_r^i < 60$	Considerable	$100 \leq RI < 200$	Considerable
$60 \leq E_r^i < 120$	High	$RI \geq 200$	High
$E_r^i \geq 120$	Very high		

Table S6 Correlation coefficients for mass fractions of 30 elements in sediments of the upper and middle course of the Raša River. Marked correlations are significant at $p < 0.05$ (N = 15).

	Li	B	Na	Mg	Al	P	K	Ca	V	Cr	Fe	Mn	Co	Ni	Cu	Zn	As	Se	Sr	Mo	Cd	Sn	Sb	Cs	Ba	Hg	Tl	Pb	U
Li	1.000																												
B	**0.940**	1.000																											
Na	**−0.668**	**−0.633**	1.000																										
Mg	**0.990**	**0.950**	**−0.608**	1.000																									
Al	**0.999**	**0.942**	**−0.645**	**0.993**	1.000																								
P	**0.988**	**0.948**	**−0.631**	**0.985**	**0.990**	1.000																							
K	**0.989**	**0.940**	**−0.567**	**0.992**	**0.993**	**0.988**	1.000																						
Ca	**−0.997**	**−0.936**	**0.640**	**−0.985**	**−0.997**	**−0.988**	**−0.992**	1.000																					
V	**0.998**	**0.943**	**−0.694**	**0.987**	**0.997**	**0.986**	**0.984**	−0.995	1.000																				
Cr	0.389	0.461	−0.015	0.429	0.408	0.508	0.464	−0.398	0.375	1.000																			
Fe	**0.943**	**0.943**	**−0.524**	**0.949**	**0.950**	**0.960**	**0.972**	**−0.955**	**0.941**	**0.535**	1.000																		
Mn	**−0.579**	**−0.566**	**0.844**	**−0.535**	**−0.560**	**−0.596**	−0.486	**0.553**	**−0.603**	−0.244	−0.431	1.000																	
Co	**0.770**	**0.805**	−0.253	**0.817**	**0.789**	**0.838**	**0.831**	**−0.776**	**0.761**	**0.764**	**0.861**	−0.388	1.000																
Ni	**0.980**	**0.945**	**−0.554**	**0.991**	**0.986**	**0.986**	**0.990**	**−0.979**	**0.976**	0.489	**0.955**	**−0.534**	**0.871**	1.000															
Cu	**0.973**	**0.927**	**−0.674**	**0.957**	**0.974**	**0.972**	**0.960**	**−0.969**	**0.977**	0.449	**0.923**	**−0.623**	**0.783**	**0.962**	1.000														
Zn	**0.993**	**0.946**	**−0.721**	**0.980**	**0.990**	**0.986**	**0.976**	**−0.990**	**0.996**	0.392	**0.941**	**−0.631**	**0.754**	**0.966**	**0.975**	1.000													
As	**0.680**	**0.783**	**−0.684**	**0.680**	**0.673**	**0.704**	**0.667**	**−0.682**	**0.695**	0.399	**0.753**	**−0.609**	**0.603**	**0.666**	**0.665**	**0.731**	1.000												
Se	−0.291	−0.291	−0.404	−0.354	−0.323	−0.370	−0.422	0.323	−0.260	**−0.726**	−0.498	−0.310	**−0.695**	−0.419	−0.278	−0.240	−0.123	1.000											
Sr	**−0.993**	**−0.929**	**0.671**	**−0.981**	**−0.991**	**−0.976**	**−0.984**	**0.992**	**−0.991**	−0.366	**−0.947**	**0.530**	**−0.739**	**−0.962**	**−0.955**	**−0.987**	**−0.682**	0.297	1.000										
Mo	**−0.785**	**−0.787**	0.287	**−0.798**	**−0.801**	**−0.848**	**−0.845**	**0.804**	**−0.773**	**−0.797**	**−0.898**	0.324	**−0.914**	**−0.842**	**−0.791**	**−0.772**	**−0.638**	**0.736**	**0.783**	1.000									
Cd	**0.947**	**0.936**	**−0.796**	**0.938**	**0.942**	**0.951**	**0.919**	**−0.933**	**0.957**	0.430	**0.890**	**−0.725**	**0.738**	**0.920**	**0.940**	**0.968**	**0.784**	−0.155	**−0.938**	**−0.741**	1.000								
Sn	**0.891**	**0.820**	**−0.687**	**0.874**	**0.885**	**0.864**	**0.860**	**−0.889**	**0.887**	0.284	**0.820**	**−0.600**	**0.608**	**0.849**	**0.859**	**0.893**	**0.710**	−0.161	**−0.880**	**−0.640**	**0.815**	1.000							
Sb	−0.070	−0.059	**−0.623**	−0.145	−0.104	−0.135	−0.206	0.102	−0.032	**−0.525**	−0.263	**−0.545**	−0.482	−0.202	−0.027	−0.005	0.115	**0.945**	0.085	0.511	0.095	0.053	1.000						
Cs	**0.999**	**0.941**	**−0.651**	**0.993**	**0.999**	**0.987**	**0.992**	**−0.997**	**0.997**	0.390	**0.948**	**−0.558**	**0.781**	**0.984**	**0.973**	**0.991**	**0.677**	−0.310	**−0.991**	**−0.790**	**0.942**	**0.888**	−0.092	1.000					
Ba	**0.978**	**0.915**	**−0.553**	**0.985**	**0.985**	**0.966**	**0.982**	**−0.973**	**0.971**	0.394	**0.926**	−0.467	**0.798**	**0.981**	**0.946**	**0.956**	**0.591**	−0.385	**−0.967**	**−0.793**	**0.899**	**0.841**	−0.197	**0.984**	1.000				
Hg	0.494	**0.532**	−0.235	0.498	0.509	0.546	0.493	−0.485	0.493	0.463	0.466	**−0.534**	**0.690**	**0.588**	**0.574**	0.469	0.253	−0.282	−0.419	**−0.532**	0.469	0.392	−0.115	0.498	**0.523**	1.000			
Tl	**0.993**	**0.945**	**−0.740**	**0.978**	**0.990**	**0.982**	**0.970**	**−0.988**	**0.996**	0.379	**0.928**	**−0.654**	**0.744**	**0.964**	**0.976**	**0.998**	**0.727**	−0.213	**−0.984**	**−0.759**	**0.967**	**0.906**	0.023	**0.990**	**0.956**	0.495	1.000		
Pb	**0.767**	**0.745**	**−0.894**	**0.712**	**0.749**	**0.759**	**0.685**	**−0.748**	**0.785**	0.274	**0.638**	**−0.911**	0.486	**0.706**	**0.823**	**0.803**	**0.680**	0.192	**−0.734**	−0.485	**0.841**	**0.778**	0.460	**0.750**	**0.660**	**0.546**	**0.827**	1.000	
U	**0.752**	**0.729**	**−0.955**	**0.700**	**0.730**	**0.706**	**0.655**	**−0.730**	**0.775**	−0.020	**0.593**	**−0.842**	0.315	**0.654**	**0.748**	**0.792**	**0.673**	0.378	**−0.740**	−0.308	**0.823**	**0.774**	**0.589**	**0.736**	**0.647**	0.337	**0.813**	**0.919**	1.000
Ag	**0.913**	**0.839**	−0.472	**0.915**	**0.921**	**0.940**	**0.932**	**−0.915**	**0.900**	**0.622**	**0.896**	−0.478	**0.853**	**0.931**	**0.909**	**0.893**	**0.539**	**−0.530**	**−0.910**	**−0.885**	**0.838**	**0.790**	−0.322	**0.911**	**0.910**	**0.581**	**0.891**	**0.656**	**0.528**

Table S7 Results of the Principal Components Analysis (PCA) (Varimax rotated) of concentration of elements in sediments from the Raša River. Significant correlations (r>0.7) are shown in bold.

Variable	Factor 1	Factor 2	Factor 3
Ag	**0.8356**	−0.0042	0.4690
Al	**0.9515**	0.1565	0.2549
As	*0.6103*	0.3684	0.2979
B	**0.8737**	0.2097	0.3325
Ba	**0.9488**	0.0518	0.2428
Ca	**−0.9552**	−0.1512	−0.2388
Cd	**0.8526**	0.3722	0.2993
Co	*0.6629*	−0.1277	**0.7051**
Cr	0.2450	−0.2024	**0.8645**
Cs	**0.9555**	0.1622	0.2362
Cu	**0.8874**	0.2466	0.3340
Fe	**0.9148**	0.0027	0.3499
Hg	0.2667	0.2294	**0.7407**
K	**0.9553**	0.0539	0.2860
Li	**0.9513**	0.1858	0.2356
Mg	**0.9494**	0.1179	0.2660
Mn	−0.3492	**−0.8315**	−0.3709
Mo	**−0.7130**	0.1829	*−0.6300*
Na	−0.5601	**−0.7871**	0.0118
Ni	**0.9175**	0.0883	0.3651
P	**0.9154**	0.1590	0.3606
Pb	0.5851	**0.7279**	0.3047
Sb	−0.1596	**0.9058**	−0.3686
Se	−0.3221	**0.7581**	−0.5524
Sn	**0.8496**	0.2892	0.1445
Sr	**−0.9685**	−0.1506	−0.1779
Tl	**0.9268**	0.2867	0.2406
U	*0.6505*	**0.7554**	−0.0256
V	**0.9454**	0.2216	0.2298
Zn	**0.9358**	0.2530	0.2369
Proportion of total variance (%)	63.0	16.0	15.1

Table S8 The enrichment factors (*EF*) of metal(loid)s in the sediments.

Location		Li	B	Na	Mg	P	K	Ca	V	Cr	Fe	Mn	Co	Ni	Cu	Zn	As	Se	Sr	Mo	Cd	Sn	Sb	Cs	Ba	Hg	Tl	Pb	U	Ag
**S2**		2.43	0.91	0.60	1.27	1.45	1.03	0.63	1.40	1.66	1.44	0.82	1.28	1.46	1.38	1.51	0.82	0.76	1.04	0.28	1.41	1.41	0.50	3.37	1.28	0.78	0.59	0.96	1.18	0.49
**S2**		2.42	0.95	0.58	1.28	1.45	1.02	0.66	1.40	1.72	1.43	0.86	1.28	1.46	1.43	1.53	0.90	0.98	1.05	0.27	1.60	1.15	0.54	3.40	1.30	0.68	0.60	0.97	1.17	0.46
**S2**		2.43	0.95	0.60	1.29	1.45	1.03	0.67	1.39	1.68	1.46	0.91	1.26	1.45	1.26	1.49	0.87	0.86	1.05	0.28	1.56	1.07	0.50	3.38	1.32	0.67	0.58	0.89	1.17	0.45
**S2**		2.44	0.96	0.62	1.31	1.42	1.04	0.68	1.38	1.71	1.49	0.99	1.23	1.44	1.28	1.51	1.03	0.71	1.07	0.30	1.39	1.71	0.48	3.39	1.31	0.55	0.59	0.93	1.18	0.46
**S2**		2.41	0.97	0.62	1.33	1.48	1.05	0.72	1.40	1.75	1.54	1.01	1.26	1.44	1.29	1.57	1.04	0.70	1.07	0.30	1.60	1.22	0.47	3.36	1.32	0.47	0.58	0.88	1.17	0.48
**S2**		2.46	0.99	0.65	1.33	1.42	1.07	0.73	1.41	1.72	1.56	1.11	1.28	1.48	1.32	1.51	1.00	0.75	1.11	0.28	1.49	1.13	0.49	3.39	1.32	0.56	0.57	0.93	1.17	0.48
**S1**		2.44	1.43	1.00	1.72	1.50	1.03	1.95	1.50	2.46	1.43	1.47	1.48	1.65	1.55	1.69	1.46	4.66	2.63	2.65	1.76	1.32	1.37	3.17	1.67	0.85	0.56	1.57	2.10	0.58
**S1**		2.40	1.23	0.97	1.64	1.41	1.07	1.76	1.44	2.31	1.45	1.62	1.52	1.62	1.44	1.55	1.21	2.64	2.33	1.92	1.57	1.24	0.97	3.26	1.67	0.67	0.50	1.28	1.65	0.57
**S1**		2.37	1.20	1.04	1.58	1.46	1.07	1.64	1.37	2.28	1.46	1.78	1.49	1.59	1.40	1.48	0.92	1.84	2.25	1.62	1.20	1.00	0.75	3.17	1.65	0.76	0.46	1.17	1.43	0.61
**S1**		2.36	1.23	1.04	1.59	1.39	1.11	1.56	1.39	2.25	1.56	1.91	1.43	1.60	1.44	1.47	0.88	1.36	2.12	1.52	1.21	1.05	0.68	3.24	1.65	0.64	0.46	1.06	1.34	0.56
**S1**		2.35	1.24	1.03	1.62	1.44	1.07	1.61	1.36	2.32	1.39	1.63	1.64	1.71	1.37	1.43	1.04	1.48	2.21	1.40	1.38	1.04	0.66	3.21	1.69	0.95	0.47	1.13	1.38	0.59
**S1**		2.38	1.24	1.10	1.66	1.53	1.13	1.66	1.39	2.62	1.48	1.66	1.72	1.73	1.42	1.52	1.03	1.22	2.35	1.42	1.39	1.00	0.69	3.20	1.65	0.75	0.46	1.17	1.36	0.60
**S1**		2.33	1.29	1.04	1.64	1.49	1.13	1.61	1.38	2.40	1.70	1.74	1.80	1.73	1.39	1.48	1.18	1.03	2.21	1.39	1.36	1.01	0.65	3.24	1.63	0.88	0.47	1.10	1.35	0.57
**S1**		2.35	1.34	1.10	1.69	1.60	1.13	1.86	1.37	3.07	1.67	1.74	1.88	1.72	1.52	1.51	1.21	0.77	2.47	0.84	1.56	1.01	0.70	3.09	1.68	0.93	0.47	1.32	1.43	0.70
**S1**		2.32	1.35	1.13	1.66	1.57	1.14	1.78	1.40	2.80	1.86	1.79	1.87	1.75	1.51	1.54	1.47	0.78	2.51	0.73	1.45	1.01	0.70	3.17	1.68	0.94	0.47	1.25	1.42	0.60
**Raša River**	Min	2.32	0.91	0.58	1.27	1.39	1.02	0.63	1.36	1.66	1.39	0.82	1.23	1.44	1.26	1.43	0.82	0.70	1.04	0.27	1.20	1.00	0.47	3.09	1.28	0.47	0.46	0.88	1.17	0.45
	Max	2.46	1.43	1.13	1.72	1.60	1.14	1.95	1.50	3.07	1.86	1.91	1.88	1.75	1.55	1.69	1.47	4.66	2.63	2.65	1.76	1.71	1.37	3.40	1.69	0.95	0.60	1.57	2.10	0.70
	AV	2.39	1.15	0.87	1.51	1.47	1.07	1.30	1.40	2.18	1.53	1.40	1.49	1.59	1.40	1.52	1.07	1.37	1.83	1.01	1.46	1.16	0.68	3.27	1.52	0.74	0.52	1.11	1.37	0.55
**S1**	Min	2.32	1.20	0.97	1.58	1.39	1.03	1.56	1.36	2.25	1.39	1.47	1.43	1.59	1.37	1.43	0.88	0.77	2.12	0.73	1.20	1.00	0.65	3.09	1.63	0.64	0.46	1.06	1.34	0.56
	Max	2.44	1.43	1.13	1.72	1.60	1.14	1.95	1.50	3.07	1.86	1.91	1.88	1.75	1.55	1.69	1.47	4.66	2.63	2.65	1.76	1.32	1.37	3.26	1.69	0.95	0.56	1.57	2.10	0.70
	AV	2.37	1.28	1.05	1.64	1.49	1.10	1.71	1.40	2.50	1.56	1.71	1.65	1.68	1.45	1.52	1.16	1.75	2.34	1.50	1.43	1.08	0.80	3.19	1.66	0.82	0.48	1.23	1.50	0.60
**S2**	Min	2.41	0.91	0.58	1.27	1.42	1.02	0.63	1.38	1.66	1.43	0.82	1.23	1.44	1.26	1.49	0.82	0.70	1.04	0.27	1.39	1.07	0.47	3.36	1.28	0.47	0.57	0.88	1.17	0.45
	Max	2.46	0.99	0.65	1.33	1.48	1.07	0.73	1.41	1.75	1.56	1.11	1.28	1.48	1.43	1.57	1.04	0.98	1.11	0.30	1.60	1.71	0.54	3.40	1.32	0.78	0.60	0.97	1.18	0.49
	AV	2.43	0.95	0.61	1.30	1.45	1.04	0.68	1.40	1.70	1.49	0.95	1.27	1.45	1.33	1.52	0.95	0.79	1.06	0.28	1.51	1.28	0.50	3.38	1.31	0.62	0.58	0.93	1.17	0.47

Table S9 Index of geo-accumulation (I_{geo}) of metal(loid)s in the sediments.

Location		Li	B	Na	Mg	P	K	Ca	V	Cr	Fe	Mn	Co	Ni	Cu	Zn	As	Se	Sr	Mo	Cd	Sn	Sb	Cs	Ba	Hg	Tl	Pb	U	Ag	Al
**S1**		−0.38	−1.14	−1.66	−0.88	−1.08	−1.62	−0.70	−1.08	−0.36	−1.15	−1.10	−1.09	−0.94	−1.03	−0.91	−1.12	0.56	−0.27	−0.26	−0.84	−1.26	−1.20	0.00	−0.92	−1.90	−2.51	−1.01	−0.59	−2.46	−1.92
**S1**		−0.29	−1.26	−1.59	−0.84	−1.05	−1.46	−0.74	−1.02	−0.34	−1.02	−0.85	−0.95	−0.86	−1.03	−0.92	−1.27	−0.15	−0.33	−0.61	−0.90	−1.24	−1.60	0.15	−0.81	−2.12	−2.54	−1.19	−0.83	−2.36	−1.81
**S1**		−0.27	−1.24	−1.45	−0.85	−0.96	−1.41	−0.80	−1.06	−0.32	−0.97	−0.68	−0.94	−0.84	−1.02	−0.95	−1.63	−0.63	−0.34	−0.81	−1.25	−1.51	−1.92	0.15	−0.79	−1.91	−2.62	−1.28	−1.00	−2.23	−1.77
**S1**		−0.22	−1.16	−1.41	−0.79	−0.99	−1.31	−0.82	−0.98	−0.29	−0.82	−0.52	−0.94	−0.78	−0.94	−0.90	−1.65	−1.01	−0.38	−0.85	−1.19	−1.39	−2.01	0.24	−0.74	−2.10	−2.58	−1.37	−1.03	−2.29	−1.72
**S1**		−0.23	−1.15	−1.43	−0.77	−0.94	−1.36	−0.78	−1.02	−0.25	−0.99	−0.76	−0.75	−0.69	−1.00	−0.95	−1.41	−0.90	−0.32	−0.98	−1.00	−1.40	−2.07	0.22	−0.71	−1.54	−2.56	−1.28	−1.00	−2.23	−1.73
**S1**		−0.27	−1.21	−1.39	−0.79	−0.91	−1.35	−0.79	−1.04	−0.13	−0.95	−0.79	−0.74	−0.73	−1.02	−0.92	−1.48	−1.24	−0.29	−1.02	−1.04	−1.52	−2.05	0.15	−0.80	−1.95	−2.63	−1.30	−1.08	−2.25	−1.78
**S1**		−0.25	−1.10	−1.41	−0.75	−0.89	−1.30	−0.79	−1.01	−0.21	−0.70	−0.67	−0.62	−0.68	−0.99	−0.90	−1.23	−1.43	−0.33	−0.99	−1.02	−1.45	−2.10	0.23	−0.76	−1.65	−2.57	−1.33	−1.03	−2.27	−1.73
**S1**		−0.37	−1.18	−1.46	−0.84	−0.92	−1.43	−0.70	−1.15	0.02	−0.86	−0.80	−0.69	−0.81	−1.00	−1.00	−1.32	−1.98	−0.29	−1.86	−0.96	−1.58	−2.11	0.03	−0.85	−1.70	−2.68	−1.20	−1.09	−2.12	−1.86
**S1**		−0.38	−1.16	−1.42	−0.86	−0.94	−1.41	−0.76	−1.11	−0.11	−0.70	−0.75	−0.69	−0.79	−0.99	−0.97	−1.03	−1.95	−0.26	−2.04	−1.05	−1.58	−2.10	0.07	−0.84	−1.68	−2.70	−1.27	−1.09	−2.32	−1.85
**S2**		0.42	−0.99	−1.61	−0.52	−0.32	−0.83	−1.52	−0.38	−0.13	−0.34	−1.15	−0.51	−0.32	−0.40	−0.27	−1.14	−1.27	−0.81	−2.71	−0.37	−0.37	−1.86	0.89	−0.51	−1.23	−1.63	−0.91	−0.62	−1.90	−1.13
**S2**		0.42	−0.94	−1.65	−0.50	−0.32	−0.82	−1.46	−0.37	−0.08	−0.34	−1.08	−0.50	−0.31	−0.33	−0.24	−1.00	−0.89	−0.79	−2.77	−0.18	−0.66	−1.73	0.91	−0.47	−1.42	−1.60	−0.90	−0.63	−1.98	−1.12
**S2**		0.41	−0.95	−1.62	−0.51	−0.34	−0.83	−1.46	−0.40	−0.13	−0.34	−1.02	−0.54	−0.34	−0.54	−0.30	−1.08	−1.10	−0.81	−2.74	−0.24	−0.78	−1.87	0.88	−0.48	−1.46	−1.67	−1.04	−0.65	−2.02	−1.14
**S2**		0.39	−0.97	−1.60	−0.51	−0.39	−0.85	−1.45	−0.44	−0.13	−0.32	−0.91	−0.60	−0.37	−0.54	−0.31	−0.85	−1.39	−0.80	−2.63	−0.42	−0.12	−1.95	0.86	−0.51	−1.75	−1.67	−1.01	−0.66	−2.01	−1.16
**S2**		0.34	−0.97	−1.62	−0.52	−0.37	−0.86	−1.39	−0.44	−0.12	−0.30	−0.92	−0.59	−0.40	−0.56	−0.27	−0.87	−1.43	−0.83	−2.68	−0.25	−0.64	−2.03	0.82	−0.53	−2.01	−1.71	−1.11	−0.70	−1.99	−1.19
**S2**		0.34	−0.97	−1.57	−0.55	−0.45	−0.86	−1.40	−0.46	−0.18	−0.31	−0.81	−0.60	−0.39	−0.55	−0.36	−0.95	−1.38	−0.81	−2.79	−0.38	−0.78	−1.98	0.81	−0.56	−1.81	−1.77	−1.07	−0.73	−2.01	−1.22
**Raša River**	Min	−0.38	−1.26	−1.66	−0.88	−1.08	−1.62	−1.52	−1.15	−0.36	−1.15	−1.15	−1.09	−0.94	−1.03	−1.00	−1.65	−1.98	−0.83	−2.79	−1.25	−1.58	−2.11	0.00	−0.92	−2.12	−2.70	−1.37	−1.09	−2.46	−1.92
	Max	0.42	−0.94	−1.39	−0.50	−0.32	−0.82	−0.70	−0.37	0.02	−0.30	−0.52	−0.50	−0.31	−0.33	−0.24	−0.85	0.56	−0.26	−0.26	−0.18	−0.12	−1.20	0.91	−0.47	−1.23	−1.60	−0.90	−0.59	−1.90	−1.12
	AV	−0.02	−1.09	−1.53	−0.70	−0.72	−1.18	−1.04	−0.80	−0.18	−0.67	−0.85	−0.72	−0.62	−0.80	−0.68	−1.20	−1.08	−0.51	−1.72	−0.74	−1.09	−1.91	0.43	−0.69	−1.75	−2.23	−1.15	−0.85	−2.16	−1.54
**S1**	Min	−0.38	−1.26	−1.66	−0.88	−1.08	−1.62	−1.52	−1.15	−0.36	−1.15	−1.15	−1.09	−0.94	−1.03	−1.00	−1.65	−1.98	−0.83	−2.79	−1.25	−1.58	−2.11	0.00	−0.92	−2.12	−2.70	−1.37	−1.09	−2.46	−1.92
	Max	0.42	−0.94	−1.39	−0.50	−0.32	−0.82	−0.70	−0.37	0.02	−0.30	−0.52	−0.50	−0.31	−0.33	−0.24	−0.85	0.56	−0.26	−0.26	−0.18	−0.12	−1.20	0.24	−0.47	−1.23	−1.60	−0.90	−0.59	−1.90	−1.12
	AV	−0.02	−1.09	−1.53	−0.70	−0.72	−1.18	−1.04	−0.80	−0.18	−0.67	−0.85	−0.72	−0.62	−0.80	−0.68	−1.20	−1.08	−0.51	−1.72	−0.74	−1.09	−1.91	0.14	−0.69	−1.75	−2.23	−1.15	−0.85	−2.16	−1.54
**S2**	Min	−0.38	−1.26	−1.66	−0.88	−1.08	−1.62	−0.82	−1.15	−0.36	−1.15	−1.10	−1.09	−0.94	−1.03	−1.00	−1.65	−1.98	−0.38	−2.04	−1.25	−1.58	−2.11	0.81	−0.92	−2.12	−2.70	−1.37	−1.09	−2.46	−1.92
	Max	−0.22	−1.10	−1.39	−0.75	−0.89	−1.30	−0.70	−0.98	0.02	−0.70	−0.52	−0.62	−0.68	−0.94	−0.90	−1.03	0.56	−0.26	−0.26	−0.84	−1.24	−1.20	0.91	−0.71	−1.54	−2.51	−1.01	−0.59	−2.12	−1.72
	AV	−0.29	−1.18	−1.47	−0.82	−0.97	−1.40	−0.76	−1.05	−0.22	−0.90	−0.77	−0.82	−0.79	−1.00	−0.94	−1.35	−0.97	−0.31	−1.05	−1.03	−1.44	−1.91	0.86	−0.80	−1.84	−2.60	−1.25	−0.97	−2.28	−1.80

Table S10 Pollution index (PLI) of metal(loid)s in the sediments.

**Location**		**C_i/B_i**																														Product Ci/Bi	**PLI (n=30)**
		**Na**	**Mg**	**P**	**K**	**Ca**	**V**	**Cr**	**Fe**	**Mn**	**Co**	**Ni**	**Cu**	**Zn**	**As**	**Sr**	**Ba**	**Hg**	**Pb**	**U**	**Al**	**Li**	**B**	**Se**	**Mo**	**Cd**	**Sn**	**Sb**	**Cs**	**Tl**	**Ag**		
S2		0.60	1.17	1.52	0.92	0.87	1.31	1.56	1.33	0.81	1.27	1.39	1.30	1.47	0.86	1.29	1.24	1.04	0.97	1.61	0.82	2.75	0.75	0.97	0.23	1.74	2.46	0.41	2.78	0.48	0.40	5.14	1.06
S2		0.59	1.18	1.52	0.92	0.90	1.32	1.62	1.34	0.85	1.27	1.39	1.36	1.49	0.95	1.31	1.27	0.91	0.99	1.59	0.83	2.76	0.78	1.26	0.22	1.99	2.02	0.45	2.82	0.49	0.38	8.30	1.07
S2		0.60	1.17	1.51	0.91	0.91	1.29	1.56	1.34	0.88	1.24	1.36	1.17	1.44	0.90	1.28	1.26	0.89	0.89	1.57	0.82	2.73	0.78	1.09	0.22	1.91	1.85	0.41	2.76	0.47	0.37	3.25	1.04
S2		0.61	1.17	1.45	0.91	0.91	1.26	1.57	1.35	0.95	1.19	1.34	1.18	1.43	1.06	1.30	1.23	0.72	0.91	1.56	0.80	2.69	0.77	0.89	0.24	1.68	2.93	0.39	2.73	0.47	0.37	3.29	1.04
S2		0.60	1.17	1.48	0.90	0.95	1.25	1.57	1.37	0.95	1.20	1.31	1.16	1.46	1.04	1.27	1.22	0.61	0.85	1.51	0.79	2.62	0.77	0.86	0.23	1.90	2.05	0.37	2.65	0.46	0.38	1.60	1.02
S2		0.62	1.14	1.40	0.90	0.94	1.24	1.51	1.36	1.02	1.19	1.32	1.17	1.37	0.99	1.28	1.20	0.70	0.88	1.49	0.77	2.61	0.76	0.90	0.22	1.73	1.86	0.38	2.62	0.44	0.37	1.19	1.01
S1		0.58	0.91	0.90	0.53	1.54	0.81	1.33	0.76	0.83	0.85	0.90	0.84	0.94	0.88	1.87	0.93	0.66	0.91	1.64	0.47	1.59	0.68	3.44	1.25	1.25	1.33	0.65	1.50	0.26	0.27	0.06	0.91
S1		0.61	0.94	0.92	0.59	1.49	0.84	1.35	0.84	0.99	0.93	0.96	0.84	0.94	0.79	1.79	1.00	0.56	0.80	1.39	0.51	1.69	0.63	2.10	0.98	1.20	1.35	0.50	1.67	0.26	0.29	0.03	0.86
S1		0.67	0.93	0.98	0.61	1.43	0.82	1.37	0.86	1.12	0.94	0.97	0.84	0.92	0.62	1.78	1.02	0.65	0.76	1.24	0.53	1.71	0.63	1.51	0.86	0.95	1.12	0.40	1.67	0.24	0.32	0.01	0.86
S1		0.69	0.97	0.96	0.66	1.41	0.86	1.40	0.96	1.24	0.94	1.01	0.90	0.94	0.61	1.73	1.06	0.57	0.71	1.20	0.55	1.77	0.67	1.16	0.83	0.99	1.22	0.37	1.77	0.25	0.31	0.01	0.87
S1		0.68	0.98	0.99	0.63	1.45	0.84	1.44	0.85	1.05	1.07	1.07	0.85	0.91	0.72	1.80	1.08	0.84	0.75	1.24	0.54	1.76	0.68	1.25	0.76	1.12	1.21	0.36	1.75	0.25	0.32	0.03	0.89
S1		0.70	0.97	1.02	0.64	1.44	0.83	1.56	0.87	1.03	1.08	1.04	0.85	0.93	0.68	1.84	1.01	0.63	0.75	1.17	0.52	1.71	0.65	0.99	0.74	1.09	1.12	0.36	1.67	0.24	0.32	0.01	0.86
S1		0.69	0.99	1.02	0.66	1.44	0.85	1.48	1.04	1.12	1.17	1.08	0.86	0.94	0.81	1.80	1.04	0.78	0.73	1.21	0.54	1.73	0.70	0.87	0.76	1.11	1.17	0.35	1.76	0.25	0.31	0.03	0.89
S1		0.67	0.93	1.00	0.61	1.53	0.77	1.73	0.93	1.02	1.12	0.98	0.86	0.88	0.76	1.84	0.98	0.75	0.80	1.16	0.50	1.60	0.66	0.59	0.41	1.16	1.07	0.35	1.53	0.23	0.34	0.00	0.83
S1		0.69	0.92	0.99	0.61	1.47	0.79	1.59	1.04	1.06	1.12	1.00	0.86	0.90	0.93	1.88	0.98	0.76	0.76	1.16	0.50	1.59	0.67	0.60	0.36	1.08	1.06	0.35	1.57	0.23	0.30	0.00	0.83
**Raša River**	Min																															0.00	0.83
	Max																															8.30	1.07
	AV																															1.53	0.94
**S1**	Min																															0.004	0.83
	Max																															0.062	0.91
	AV																															0.021	0.87
**S2**	Min																															1.19	1.01
	Max																															8.30	1.07
	AV																															3.80	1.04

Table S11 E_r^i and *RI* of metal(loid)s in the sediments

Location		E_r^i												RI
		V	Cr	Mn	Co	Ni	Cu	Zn	As	Ba	Hg	Pb	Cd	
**S1**		1.61	2.67	0.83	4.23	4.51	4.19	0.94	8.79	0.93	26.2	4.56	37.6	97
**S1**		1.68	2.70	0.99	4.67	4.78	4.20	0.94	7.89	1.00	22.4	4.02	36.1	91
**S1**		1.64	2.74	1.12	4.71	4.84	4.22	0.92	6.17	1.02	26.0	3.78	28.5	86
**S1**		1.72	2.81	1.24	4.69	5.03	4.48	0.94	6.10	1.06	22.7	3.54	29.6	84
**S1**		1.68	2.88	1.05	5.35	5.35	4.27	0.91	7.21	1.08	33.7	3.77	33.7	101
**S1**		1.66	3.12	1.03	5.38	5.20	4.23	0.93	6.84	1.01	25.3	3.73	32.7	91
**S1**		1.70	2.97	1.12	5.87	5.41	4.32	0.94	8.13	1.04	31.0	3.64	33.2	99
**S1**		1.54	3.46	1.02	5.58	4.92	4.29	0.88	7.62	0.98	30.1	4.00	34.7	99
**S1**		1.58	3.17	1.06	5.60	5.01	4.30	0.90	9.33	0.98	30.4	3.81	32.5	99
**S2**		2.63	3.12	0.81	6.34	6.95	6.48	1.47	8.64	1.24	41.6	4.87	52.2	136
**S2**		2.65	3.25	0.85	6.37	6.96	6.79	1.49	9.52	1.27	36.6	4.93	59.8	140
**S2**		2.59	3.13	0.88	6.19	6.81	5.87	1.44	9.04	1.26	35.6	4.47	57.2	134
**S2**		2.52	3.13	0.95	5.96	6.69	5.88	1.43	10.6	1.23	29.0	4.57	50.4	122
**S2**		2.51	3.14	0.95	6.00	6.55	5.80	1.46	10.5	1.22	24.3	4.25	56.9	124
**S2**		2.48	3.02	1.02	5.97	6.59	5.84	1.37	9.87	1.20	27.9	4.38	51.9	122
**Raša River**	Min	1.54	2.67	0.81	4.23	4.51	4.19	0.88	6.10	0.93	22.4	3.54	28.5	84
	Max	2.65	3.46	1.24	6.37	6.96	6.79	1.49	10.6	1.27	41.6	4.93	59.8	140
	AV	2.01	3.02	0.99	5.53	5.71	5.01	1.13	8.41	1.10	29.5	4.15	41.8	108
**S1**	Min	1.54	2.67	0.83	4.23	4.51	4.19	0.88	6.10	0.93	22.4	3.54	28.5	84
	Max	1.72	3.46	1.24	5.87	5.41	4.48	0.94	9.33	1.08	33.7	4.56	37.6	101
	AV	1.65	2.95	1.05	5.12	5.01	4.28	0.92	7.57	1.01	27.6	3.87	33.2	94
**S2**	Min	2.48	3.02	0.81	5.96	6.55	5.80	1.37	8.64	1.20	24.3	4.25	50.4	122
	Max	2.65	3.25	1.02	6.37	6.96	6.79	1.49	10.6	1.27	41.6	4.93	59.8	140
	AV	2.56	3.13	0.91	6.14	6.76	6.11	1.44	9.68	1.24	32.5	4.58	54.7	130

Table S12 Comparison of average concentrations [arithmetic mean (range) or arithmetic mean ± standard deviation] of potentially toxic elements [in mg kg^{-1} or µg kg^{-1} wet mass (wm)] in muscle tissue of six freshwater fish species from the Raša River (Istria, Croatia) with the literature data for similar freshwater species from European rivers, lakes and reservoirs

Species	Country	Location	As (mg kg^{-1})	Cd (µg kg^{-1})	Co (mg kg^{-1})	Cr (µg kg^{-1})	Cu (mg kg^{-1})	Hg (mg kg^{-1})	Mn (mg kg^{-1})	Ni (µg kg^{-1})	Pb (µg kg^{-1})	Se (mg kg^{-1})	Zn (mg kg^{-1})	Reference
Eel														
European eel	UK	Mersey estuary	1.1 (0.14 -				1.06 (0.22–4.1	1.33 (0.15–2.5			2.2 (0.3–4.7)		25.7 (16.0–32.	[80]
		57 sites						0.24–0.39						[81]
		River Yare						0.26 (0.06–0.5						[82]
		Ormesby Broad						0.10 (0.02–0.2						
	Belgium	Yser	0.14	2.45		296	0.52	0.15		46.52	41.7	0.329	23.9	[27]
		Meuse	0.37	19.5		362	0.49	0.17		65.7	37.6	0.664	26.3	
		Scheldt	0.31	2.99		174	0.64	0.09		46.5	52.8	1.02	25.1	
		357 locations		11.7 (<DL -				114 (0.01-		186 (<DL –	57 (<DL –			[83]
		365 locations	0.17 (0.01–1.81)	15.8 (1.0–2,474)		255 (17.5–13,690)	0.91 (0.05–436)	0.12 (0.01–1.19)		208 (5.0–16,300)	81.2 (1.0–3,453)	0.75 (0.03–5.10)	25.9 (1.2–243)	[84]
		11 river basins	0.095 (0.01–1.88)	7.7 (0.25–554)		200 (45.5–6,155)	0.46 (0.06–6.57)	0.10 (0.01–0.71)		57 (1.16–16,300)	23.6 (1.00–669)	0.66 (0.10–4.34)	20.2 (1.20–243)	[85]
		23 locations	0.08±0.01	4.3±0.2	9.7±1.1	44.7±9.7	0.13±0.04	0.15±0.01	0.2±0.0	179±14.7	62.3±1.4		16.5±0.5	[86]
	Germa	Rhine						0.05–0.4						[87]
	Poland	5 regions	0.03–0.78	1–46			0.12–0.51	0.10–0.49			<DL–28	0.10–0.31	15.9–32.0	[88]
	Latvia	5 lakes	0.02–0.44	4–15		230–550	0.58–1.27	0.071–0.64	0.11–0.60	40–270	4–84	0.27–0.56	24–47	[89]
	Spain	Turia River	0.228 (0.024–0.993)	4.9 (2.96–7.92)			0.977 (0.222–0.977)				102 (22.3–258)		17.0 (12.6–17.0)	[51]
		Odiel. Cadiz	0.52–2.91	15–50		143–368	0.5–1.5	0.010–0.023	4.71–14.1	15–20	30–90		10.1–13.0	[55]
		wild ecosystem		1–147			0.122–0.444	0.110–0.803			0.95–204			[90]
		Albufera Lake		<DL			0.18–0.33	0.02–0.24	0.10–1.46		20–230		12.8–36.8	[91]
		Mar Menor Lagoon		2 (<DL–47)				0.01 (<DL–0.18)			93 (<DL–1,434)	0.30 (<DL–1.54)		[92]
	Portugal	Ria de Aveiro		8.98–42.			0.637–1.			157–394	44.0–78.		14.0–23.	[54]
			1.27 (0.39–3.07)	3.0 (1.0–11.0)		738 (382–1,527)	0.22 (0.13–0.57)	0.14 (0.06–0.29)		156 (85–283)	23 (8–96)		13.9 (8.85–21.6)	[93]
	France	Gironde Estuary		<LOD			0.15±0.02	0.17±0.02					10.2±1.0	[52]
		Nat. Reserve	38.1–1,586	ND	2.38–16.7	336–869	0.04–0.12	0.03–0.16	0.02–0.10	14.3–293	45.2–326		2.95–14.7	[94]

Table S12 (continued)

Species	Country	Location	As (mg	Cd (µg kg^{-1})	Co (mg	Cr (µg kg^{-1})	Cu (mg	Hg (mg	Mn (mg	Ni (µg kg^{-1})	Pb (µg kg^{-1})	Se (mg	Zn (mg	Reference
European eel	France	5 fishing areas	0.10 (0.03–0.65)	11 (1–71)				0.20 (0.05–0.59)			24 (1–159)			[46]
	Italy	Vargano Lagoon	0.5	70–80			0.4–0.5	0.01–0.02			30–50		46.0–47.7	[95]
		Lago di Garda						0.23 (0.07–0.47)		410 (10–670)	500 (1–1,220)			[96]
		Fogliano Lagoon					0.29	0.31					30.4	[97]

							(0.09–0.49)	(0.08–0.57)					(18.0–47.2)	
		Caprolace Lagoon					0.31 (0.17–0.54)	0.30 (0.07–0.72)					28.9 (19.2–51.2)	
	Turkey	Asi River		70±30		60±30	0.9±0.18	0.26±0.1	0.29±0.		560±120		12.5±1.3	[98]
	Hungary	Lake Balaton		570–1,240			2.18–2.38	0.07–0.12			360–410		97.3–105	[99]
	B&H	NP Hutovo Blato	0.09 (0.08–0.10)	20 (16–26)				0.16 (0.15–0.16)			123 (117–128)			[53]
	Montenegro	Lake Skadar	0.29 (0.17–0.38)	<DL	<DL	69 (50–102)	0.84 (0.70–1.00)	0.14 (0.11–0.21)	0.64 (0.55–0.72)	38 (20–100)	<DL		15.1 (12.7–19.0)	[47]
	Croatia, B&H	Neretva River	0.054											[57]
	Croatia	River Raša	0.04 (0.02–0.08)	2.58 (1.14–5.41)	0.51 (0.02–10.5)	14.4 (3.06–31.1)	0.22 (0.16–0.32)	0.23 (0.11–0.73)	0.28 (0.16–0.63)	20.4 (7.2–70.5)	2.94 (0.60–18.0)	1.17 (0.58–1.54)	17.7 (11.5–27.7)	***This study***
Carp														
Prussian carp	Slovenia	Velenjsko Lake		<DL				0.14 (0.05–0.31)			20 (<DL–40)		6.71 (0.05–10.9)	
	Slovenia	Šalek Lakes	0.03 (<DL–0.07)	10 (<DL–10)				0.14 (0.05–0.31)			20 (<DL–40)		6.71 (0.05–10.9)	[100]
	B&H	NP Hutovo Blato	0.01 (0.007–0.014)	10 (7–14)				0.15 (0.14–0.15)			100 (94–105)			[101]
	Croatia, B&H	Neretva River	0.352											[53]
	Croatia	Raša River	0.05 (0.04–0.05)	0.15 (0.15–0.16)	4.51 (0.06–8.96)	12.0 (2–21)	0.31 (0.22–0.40)	0.42 (0.20–0.64)	0.27 (0.18–0.37)	29.8 (5.60–54.1)	4.65 (3.61–5.70)	0.91 (0.88–0.94)	7.88 (6.68–9.09)	[57]
Barbel														***This study***
Common	Spain	Turia River	0.02 (0.01–0.	1.80 (0.11–4.3			0.79 (0.21–6.2				62 (102–312		3.60 (2.21–5.8	
Danube barbel	Slovenia	Šalek Lakes	0.05 (0.02–0.11)	20 (<DL–150)				0.1 (0.06–0.15)			20 (<DL–40)		11.3 (8.74–14.0)	[51]
	NE Italy	Barbucina creek	4	7	0.11	200	5.28	0.55	18.78	230	100	0.8	34	[101]
W. Balkan barbel	Rep. N Macedonia	Crn Drim River		2.1–8.4			0.4–1.6		0.5–0.9		100–400			[102]

Table S12 (continued)

Species	Country	Location	As (mg kg^{-1})	Cd (μg kg^{-1})	Co (mg kg^{-1})	Cr (μg kg^{-1})	Cu (mg kg^{-1})	Hg (mg kg^{-1})	Mn (mg kg^{-1})	Ni (μg kg^{-1})	Pb (μg kg^{-1})	Se (mg kg^{-1})	Zn (mg kg^{-1})	Reference
Common barbel	Serbia	Danube River	0.35±0.03		0.02±0.01	91.1±13.3	0.42±0.07	0.06±0.05	0.20±0.04	42.2±8.9	24.4±24.4	0.12±0.04	4.08±0.67	[103]
		West Morava	0.21±0.05		0.01±0.00	97.7±13.3	0.43±0.03	0.02±0.01	0.27±0.06	33.3±6.6	20.0±20.0	0.07±0.02	6.69±0.43	
		South Morava	0.16±0.04		0.01±0.00	80.0±8.88	0.38±0.04	0.08±0.02	0.28±0.05	24.4±4.4	37.8±37.8	0.14±0.05	5.52±0.61	
Italian barbel	Croatia	Raša River	0.06 (0.02–0.12)	0.38 (0.20–0.58)	0.04 (0.01–0.11)	1.0 (0.2–9)	0.31 (0.19–0.44)	0.14 (0.10–0.24)	0.49 (0.42–0.63)	7.12 (3.12–9.62)	1.80 (0.45–2.11)	0.70 (0.42–1.22)	5.68 (4.40–6.85)	***This study***
Roach														
	UK	2 locations						0.02–0.12						[82]

Common roach	France	5 French fishing areas	0.09 (0.03–0.26)	5 (1–50)				0.09 (0.04–0.30)			59 (1–340)			[46]
		Seine River		15–115		250–325	0.45–0.48		1.2–1.4		1,000–1,200		18–30	[104]
		Seine River		135		15	0.95		1.08		15		21	[105]
	Czech Republi	Elbe River						0.09–0.45						[106]
	Slovenia	Velenjsko Lake		<DL				0.08 (0.03–0.16)			30 (10–50)		13.4 (5.25–22.2)	[100]
		Šalek Lakes	0.07 (0.04–0.10)	<DL				0.08 (0.03–0.16)			30 (10–50)		13.4 (5.25–22.2)	[101]
	Poland	Lake Pluszne					0.26 (0.17–0.39)	0.07 (0.06–0.10)					6.12 (4.08–7.78)	[107]
		Warmia and Mazury Region					0.21±0.13	0.14±0.03	0.11±0.05				4.52±1.04	[108]
Albanian roach	Montenegro	Skadar Lake	0.06 (<DL–0.15)	<DL	<DL	178 (70–310)	0.57 (0.38–0.92)	0.10 (0.05–0.14)	5.12 (0.37–9.4)	41 (20–60)	<DL		19.5 (7.77–34.8)	[47]
Adriatic roach	Croatia	Raša River	0.07 (0.06–0.08)	1.00 (0.68–1.23)	0.07 (0.02–0.15)	4 (2–8)	0.25 (0.21–0.39)	0.11 (0.08–0.14)	2.41 (0.49–4.06)	21.9 (10.7–30.1)	11.3 (1.21–14.8)	0.74 (0.70–0.80)	21.9 (11.9–25.7)	*This study*
Chub														
E. chub	Serbia	Pestan River	<LOD–0.22			2.22–95.6	0.20–0.39	0.20–0.40	0.18–0.23	<LOD–82.2	<LOD–77.7		7.24–16.5	[76]
		Beljanica River	0.04–0.13			4.44–57.8	0.26–0.49	0.12–0.23	0.17–0.44	2.22–82.2	<LOD–51.1		8.9–16.1	
	Croatia	NP Plitvice Lakes	0.02 (0.01–0.03)	2.1 (0.55–5.4)	0.003 (0.002–0.005)	8.1 (1–21)	0.32 (0.20–0.45)	0.09 (0.02–0.56)	0.21 (0.11–0.38)	11.6 (3–27)	4.1 (1–14)	0.30 (0.14–0.54)	8.2 (4.3–12.5)	[19]
Italian chub	Croatia	Raša River	0.03 (0.02–0.06)	0.86 (0.37–1.33)	0.04 (0.003–0.71)	3 (1–17)	0.34 (0.17–0.42)	0.08 (0.04–0.19)	0.51 (0.21–2.17)	9.08 (1.94–23.6)	1.22 (0.63–3.59)	0.60 (0.37–0.80)	11.0 (6.78–20.0)	*This study*

Table S12 (continued)

Species	Country	Location	As (mg	Cd (µg kg⁻¹)	Co (mg	Cr (µg kg⁻¹)	Cu (mg	Hg (mg	Mn (mg	Ni (µg kg⁻¹)	Pb (µg kg⁻¹)	Se (mg	Zn (mg	Reference
Grey mullet														
Thicklipped grey mullet	Italy	Adriatic Sea				160±50	0.84±0.17			1,220±250	40±20		6.90±0.16	[109]
		Tyrrhenian Sea		<DL		<DL–19	0.34±0.20	0.10±0.06			<DL–11		6.11±2.54	[110]
		Tyrrhenian Sea		<DL–5		<DL–15	0.73±1.01	0.05±0.04			<DL–24		5.46±1.86	
	Portugal	Ria de Aveiro		6.32–7.30			0.85–0.86			85.1–98.5	53.0–61.2		9.47–10.8	[54]
Thinlipped	Italy	Adriatic Sea				150±60	0.88±0.06			1,100±240	50±20		6.53±1.34	[109]
Golden grey mullet	Italy	Straits of Messina.		61.4 (30.2–90.		292 (190–421	2.80 (2.00–3.8			294 (150–371	386 (280–550		7.31 (6.17–9.0	[111]
		Adriatic Sea				150±60	0.93±0.14			1,060±120	40±20		6.59±1.14	[109]
	Spain	Atlantic coast	1.38–2.00	13–30		29–38	0.2–0.6	0.010–0.013	2.25–2.50	21–70	30–50		3.10–8.41	[55]
	Portugal	Ria de Aveiro		23.6–25.9			0.74–0.84			93.2–111	82.3–94.7		11.6–12.2	[54]
	France	La Plata		<LOD				0.40±0.0[illegible]					49±4	[52]

Lebranche mullet		Guadalqu[illegible]		<LOD			0. 5±0.3						8.5±5.9	
		Gironde estuary		<LOD			0.34±0.05	0.10±0.01					4.2±0.5	
Flathead grey mullet	Portugal	Ria de Aveiro		1.83–20.1			0.49–0.64			57.3–107	4.05–44.1		4.63–7.95	[54]
	Algeria	Annaba [illegible]		106			1.8			55.1	269		24.0	[112]
	Bulgaria	Varna, Black Sea	0.90±0.10	24±2				0.08±0.01			70±10			[113]
		Nesebar, Black Sea	1.10±0.10	12±2				0.05±0.01			50±10			
	Turkey	Iskenderun Bay		8.88–29.3									5.82–27.6	[114]
		Iskenderu[illegible]				1,460±53	1.45±0.6			1,220±80	7,450±3,[illegible]		38.2±14.[illegible]	[115]
	Tunisia	Gulf of Gabes		933±622			1.39±0.85				1,157±318		0.17±0.02	[116]
	Croatia	Neretva River	0.31±0.05 (0.26–0.42)	52±30 (14–100)				0.20±0.04 (0.12–0.25)			106±6 (100–115)			[56]
	Croatia, B&H	Neretva River	0.33											[57]
	Croatia	Raša Bay	0.36–1.3	0.9–1.3	0.005–0.008	31–106	0.24–0.41	0.001–0.041	0.13–0.35	42–599	2–17	0.31–0.53	4.6–5.1	[9]
	Croatia	Raša River	0.42 (0.36–0.71)	0.31 (0.25–0.37)	0.01 (0.01–0.03)	4 (3–9)	0.37 (0.26–0.67)	0.04 (0.02–0.06)	0.17 (0.12–0.37)	8.22 (5.31–9.48)	3.54 (0.85–5.06)	0.27 (0.23–0.59)	7.78 (7.10–10.0)	***This study***

Table S13 Comparison of average concentrations [arithmetic mean (range) or arithmetic mean ±standard deviation] of macro (Ca, K, Mg, Na) and trace (Ag, Fe, Mo, Sr, Tl, V) elements [in mg kg^{-1} or μg kg^{-1} wet mass (wm)] in muscle tissue of six freshwater fish species from the Raša River (Istria, Croatia) with the literature data for similar freshwater species from European rivers, lakes and reservoirs

**Species**	**Country**	**Location**	**Ca (mg**	**K (mg**	**Mg (mg**	**Na (mg**	**Ag (μg**	**Fe (mg**	**Mo (μg**	**Sr (mg**	**Tl (μg**	**V (μg**	**Reference**
Eel													
European eel	Croatia	Raša River	221 (108–346)	2,429 (1,831–3,002)	193 (140–241)	545 (387–695)	0.65 (0.08–4.75)	2.91 (1.83–4.60)	2.44 (0.13–4.45)	0.38 (0.12–0.82)	2.56 (0.81–5.21)	2.78 (1.05–5.01)	***This study***
	Belgium	Belgium [illegible]					18.3±0.[illegible]	2.1±0.3	32.1±4.2		4.4±0.6	5.0±0.5	[86]
	Poland	5 regions	142–864	2,046–2,599	158–267	463–739		2.37–13.1		0.06–6.31			[88]
	Latvia	5 lakes			161–240			6.1–17.4		0.04–0.34			[89]
	Spain	Odiel, [illegible]						4.11–5.[illegible]					[55]
	Spain	Albufera [illegible]						3.72–1[illegible]					[91]
	France	Camargue Biosphere			119–181	57.6–332		2.60–6.24	4.76–21.4	0.25–0.72			[94]
	Turkey	Asi River						14.1±3.[illegible]					[98]
	Montenegro	Skadar Lake	5.31±1.95 [illegible]		1.84±1.23 [illegible]			3.23±0.78 [illegible]					[47]
Barbel													
Italian barbel	Croatia	Raša River	447 (397–683)	2,690 (2,562–3,328)	265 (246–280)	425 (309–483)	0.16 (0.08–1.95)	2.62 (2.22–3.96)	2.75 (1.47–4.09)	0.66 (0.59–1.16)	1.10 (0.44–2.03)	3.21 (2.07–5.56)	***This study***
Danube barbel	NE Italy	Barbucina Creek						85.1	80		2	1,400	[102]
W. Balkan	N Macedon	Crn Drim River						4.6–10.4					[74]
Common barbel	Serbia	Danube River							40.0±11.1	0.18±0.07			[103]
	Serbia	West Morava							28.9±17.8	0.52±0.12			

Species	Country	Location	Ca (mg	K (mg	Mg (mg	Na (mg	Ag (µg	Fe (mg	Mo (µg	Sr (mg	Tl (µg	V (µg	Reference
	Serbia	South Morava							2.22±22.2	1.30±0.19			
Roach													
Adriatic roach	Croatia	Raša River	3,944 (531–4,	2,639 (2,481–2,	306 (250–3	425 (411–4	0.36 (0.12–1	3.79 (2.83–5	9.57 (6.44–10	7.59 (0.97–8.	1.03 (0.51–1.	8.87 (2.12–1	*This study*
Common roach	France	Seine River						16.5–2					[104]
	France	Seine River						16					[105]
	Poland	Warmia and Mazury						1.01±0.44					[108]
Albanian roach	Montenegro	Skadar Lake	38.5±27.9		9.3±5.1 (2.6–16			14.5±7.41					[47]
**Table S13** (continued)													
**Species**	**Country**	**Location**	**Ca (mg**	**K (mg**	**Mg (mg**	**Na (mg**	**Ag (µg**	**Fe (mg**	**Mo (µg**	**Sr (mg**	**Tl (µg**	**V (µg**	**Reference**
Chub													
Italian chub	Croatia	Raša river	485 (171–3,855)	3,234 (1,236–3,539)	250 (148–337)	377 (183–403)	0.20 (0.10–0.92)	3.36 (1.96–4.84)	4.77 (2.90–7.45)	0.81 (0.34–4.80)	0.80 (0.38–1.47)	3.20 (1.13–7.59)	**This study**
	Hungary	River Szamos/Someş	151–307	1,063–3,070	105–264	186–364		1.58–8.27		0.33–1.25			[117]
European chub	Serbia	Pestan River						1.57–2.44	6.67–136	0.22–0.68			[76]
	Serbia	Beljanica River						1.69–3.91	15.6–42.2	0.4–4.0			[76]
	Croatia	National Park Plitvice Lakes	525 (119–1,676)	4,556 (4,105–5,028)	356 (318–404)	672 (560–867)	0.14 (0.03–0.39)	5.33 (2.89–7.52)	4 (1–27)	0.17 (0.04–0.39)	1.80 (0.70–3.61)	9 (7–16)	[19]
Grey mullet													
Flathead grey mullet	Croatia	Raša River	137 (92–316)	3,524 (3,248–3,727)	244 (203–256)	351 (268–367)	0.17 (0.10–0.37)	8.16 (5.17–10.2)	29.8 (2.91–89.4)	0.50 (0.22–1.76)	4.22 (0.43–8.93)	4.50 (2.87–9.46)	**This study**
	Algeria	Annaba Gulf						32.8					[112]
	Turkey	Iskenderun Bay						0.73–2.27					[114]
	Turkey	Iskenderun Bay						70.3 ± 0.4					[118]
	India	Krishna River	171–217	2,260–2,553	231–246	318–402		6.8–7.3					[119]
	Italy	St. Giusta Lagoon, Sardinia	86.5–473	3,000–5,560	240–393	188–488		2.5–16.0					[120]
	Croatia	Raša Bay					0.4–1.5	6.5–10.5	3.8–14			7.3–14.0	[9]
Golden grey mullet	Spain	Atlantic coast						4.11–7.13					[55]
Carp													
Prussian carp	Croatia	Raša River	348 (223–4	2,744 (2,562–2,	258 (236–2	306 (276–3	0.23 (0.20–0	5.58 (4.49–6	2.40 (2.12–2.	0.45 (0.30–0.	0.39 (0.24–0.	1.60 (1.31–1.	*This study*
European carp	Croatia	Croatian market						7.4 (2.6–11					[121]
	Montenegro	Skadar Lake	6.08 (4.1–13		2.33 (0.3–4.			6.95 (2.62–1					[47]
Common carp	Turkey	3 water reservoirs	120–374	2,956–4,164	208–297	211–353							[122]
	Bulgaria	different production	287±15	3,406±76	325±8	344±16		5.86±0.38					[123]
Crucian carp	Poland	fish farms	1,030±250	2,810±240	250±270	350±530							[124]
Silver carp	Poland	fish farms	290±370	2,920±530	230±210	270±210							
